# Industrial Needs in the Fields of Artificial Intelligence, Internet of Things and Edge Computing

**DOI:** 10.3390/s22124501

**Published:** 2022-06-14

**Authors:** Dorota Stadnicka, Jarosław Sęp, Riccardo Amadio, Daniele Mazzei, Marios Tyrovolas, Chrysostomos Stylios, Anna Carreras-Coch, Juan Alfonso Merino, Tomasz Żabiński, Joan Navarro

**Affiliations:** 1 Faculty of Mechanical Engineering and Aeronautics, Rzeszów University of Technology, 35-959 Rzeszow, Poland; dorota.stadnicka@prz.edu.pl (D.S.); jsztmiop@prz.edu.pl (J.S.); 2 Computer Science Department, University of Pisa, 56127 Pisa, Italy; r.amadio@studenti.unipi.it (R.A.); daniele.mazzei@unipi.it (D.M.); 3 Laboratory of Knowledge and Intelligent Computing, Department of Informatics and Telecommunications, University of Ioannina, 47150 Arta, Greece; tirovolas@kic.uoi.gr (M.T.); stylios@isi.gr (C.S.); 4 Industrial Systems Institute, Athena Research Center, 26504 Patras, Greece; 5 Research Group in Internet Technologies & Storage, La Salle Campus Barcelona, Universitat Ramon Llull, 08022 Barcelona, Spain; jnavarro@salleurl.edu; 6 Systems Department (257), Elecnor Servicios y Proyectos S.A.U., Carrer d’Antonio de los Rios Rosas, 40, 08940 Cornellà de Llobregat, Spain; juanalfonso.merino@elecnor.es; 7 Faculty of Electrical and Computer Engineering, Rzeszów University of Technology, 35-959 Rzeszow, Poland; tomz@prz.edu.pl

**Keywords:** Internet of Things, Artificial Intelligence, Edge Computing, industrial problems, industrial needs, Industry 4.0

## Abstract

Industry 4.0 corresponds to the Fourth Industrial Revolution, resulting from technological innovation and research multidisciplinary advances. Researchers aim to contribute to the digital transformation of the manufacturing ecosystem both in theory and mainly in practice by identifying the real problems that the industry faces. Researchers focus on providing practical solutions using technologies such as the Industrial Internet of Things (IoT), Artificial Intelligence (AI), and Edge Computing (EC). On the other hand, universities educate young engineers and researchers by formulating a curriculum that prepares graduates for the industrial market. This research aimed to investigate and identify the industry’s current problems and needs from an educational perspective. The research methodology is based on preparing a focused questionnaire resulting from an extensive recent literature review used to interview representatives from 70 enterprises operating in 25 countries. The produced empirical data revealed (1) the kind of data and business management systems that companies have implemented to advance the digitalization of their processes, (2) the industries’ main problems and what technologies (could be) implemented to address them, and (3) what are the primary industrial needs and how they can be met to facilitate their digitization. The main conclusion is that there is a need to develop a taxonomy that shall include industrial problems and their technological solutions. Moreover, the educational needs of engineers and researchers with current knowledge and advanced skills were underlined.

## 1. Introduction

The term Industry 4.0 (I4.0), also referred to as the Fourth Industrial Revolution [1], can describe the digitalization and automation of manufacturing technologies to increase the flexibility, responsiveness, and adoption of data-driven decisions in fabrication processes [2]. Differently from the Third Industrial Revolution (also referred to as Digital Revolution [1]), Industry 4.0 fosters the adoption of Cyber-Physical Systems (CPS) in the manufacturing process, which can be seen as digital assets able to operate and interact autonomously (or with little human intervention) between each other [2]. Hence, since 2011—when this term was first coined by the German government—Industry 4.0 promises to bring an unprecedented shift in the way production and supply chains that were conceived until the late 1990s and early 2000s [3,4].

This massive shift is supposed to be fueled by the latest advances in Information and Communication Technologies (ICTs), impacting and changing the way we live our daily lives [5]. In fact, the ever-growing potential of digital technologies has widened the horizons of what is doable in today’s industry: ranging from modern control systems able to collect and process vast amounts of real-time data to adjusting the production process and improving the product quality [6], to new machinery for distributed manufacturing that takes advantage of communications networks to optimize the production processes [7], including techniques to reduce energy consumption and greenhouse gas emissions in factories [8] or to reduce the downtime of production pipelines by applying predictive maintenance strategies [9].

Unavoidably, all these developments have a common background: data. Indeed, the digitalization of manufacturing processes implicitly brings an exponential growth of data [10]. That is, the more digital devices in the manufacturing process, the more data are generated and impact the process itself. Effectively, modern trends in the industry (i.e., Industry 4.0) aim to squeeze digital assets to collect as much data as possible [9], enabling practitioners to implement the novel added value services mentioned above. Therefore, the industry has a latent technological need to facilitate all the data processing needed for decision-making. This need can be articulated into three main technological pillars: Internet of Things (IoT)—also referred to as Industrial IoT (IIoT) when applied in the scope of industrial environments, Edge Computing (EC), and Artificial Intelligence (AI)— the three major technological drivers that support Industry 4.0 [2,9,11].

First, the IoT provides the required physical means to interconnect and integrate a deluge of heterogeneous devices in a simple yet effective way [12]. Thanks to the IoT, devices with modest data storage and processing capabilities can operate autonomously and, if necessary, take advantage of the computing power of neighboring devices. For instance, this becomes paramount to implementing autonomous Machine-to-Machine (M2M) communications [13]. Second, EC provides architectural guidance to reduce response time and optimize digital assets’ storage and computing capabilities spread over the factory [14]. Specifically, EC proposes distributing the computation process on the edge devices that are physically close to where data were generated rather than sending the whole bulk of data to a far entity (i.e., cloud). Thus, exploiting data locality allows for improving the available communications bandwidth of the network. Additionally, limiting the scope of data (i.e., the number of devices in which a datum has been processed) improves the cybersecurity of the manufacturing system. Finally, AI enables practitioners to automatically analyze and extract actionable insights (e.g., engine’s health status, anomalies detection, products’ quality, etc.) from all generated data through knowledge-based models [15]. These models are built from available historical data to explain (and forecast) the hidden underlying relations between different events in the manufacturing process [9].

Due to the continuous advances occurring in these three pillars day-to-day, it is safe to consider that these technologies are mature enough to be implemented in industrial environments [2]. In fact, there are a considerable amount of (success) stories and use cases in the literature describing the adoption of these technologies to make Industry 4.0 a reality [16]. However, many companies still struggle to take advantage of IoT, EC, and AI to improve and modernize their manufacturing process [17].

This research aims to discover the main technological barriers—in terms of both technology and training—in the fields of IoT, EC, and AI that industrial companies face nowadays and prevent them from transforming Industry 4.0 promises into reality. Specifically, this work links the barriers companies face nowadays to adopting Industry 4.0 standards with the real-world problems that motivate them. Additionally, it discusses the training needs in these fields that the industrial practitioners may have. As a matter of fact, this work materializes the experiences and knowledge gained in the context of the Erasmus+ project “Practical Learning of Artificial Intelligence on the Edge for Industry 4.0” (PLANET4). Collected data from 70 enterprises operating almost all over the world are used to elaborate training recommendations for industrial practitioners.

Overall, the contributions of this work are the following:

1. An analysis on which are the current enterprise management systems that production companies use nowadays and up to what extent they use data transfer automatization and their associated functionalities;
2. An analysis of companies’ needs and challenges in terms of both training and technology in the fields of IoT/IIoT, EC, and AI to successfully implement the Industry 4.0 vision;
3. An analysis of the practical skills that industrial workers should have in the fields of IoT, EC, and AI;
4. A starting point for the development of training curricula to help industrial companies to fill the identified skill gaps and, thus, enable them to address the identified challenges.

A critical difference between this research work and the existing ones is that it does not focus on industries of a specific country but includes companies from all over the world to ensure the reliability of the results. The fewer countries included in the study, the greater the probability the results will be biased since there are significant country-to-country differences in digitization and implementation of the Industry 4.0 vision. Furthermore, this research takes a more holistic approach, trying to discover the challenges at all manufacturing process phases.

The remainder of this paper is organized as follows. Section 2 presents a literature review on industrial companies’ challenges and needs in the fields of AI, IoT, and EC. Section 3 details the selected methodology to conduct this work. Section 4 presents the obtained results from the companies’ surveys. In Section 5, the questionnaire results are statistically analyzed. Section 6 presents the results of the interviews. Section 7 discusses the main findings discovered in this research. Finally, Section 8 concludes the paper.

## 2. The State of the Art about the Needs of the Industry of the Future

The literature review aims to discover the approaches used in industrial needs identification and the industrial needs presented in the literature in the field of AI, IoT, and EC. 

The article [18] highlights how there have been few scientific insights in recent years regarding the implementation of Industry 4.0 in small and medium-sized enterprises (SMEs). Starting from the state of the art of the literature, the article’s author divides the problems into challenges and research gaps. The challenges focus more on the business aspect like the financial costs, the formation of the workforce, and the application of production management models. The research gaps, in turn, can be divided into two issues: models and frameworks of Industry 4.0 applicable to SMEs because most of them have been designed for Multi-National Enterprise (MNE). The second research issue concerns evaluating the impact of I4.0 technologies on the companies that adopt them to assess the benefits and costs. 

The article [19] focuses on the technological and strategic challenges of Industry 4.0, specifically Cyber Physical System (CPS). The article defines a list of objectives to use as guidelines for implementing I4.0 technologies:

1. Standardization of systems and building a reference architecture;
2. Efficient management;
3. Establishment of a comprehensive and reliable industrial broadband infrastructure;
4. Safety and security;
5. Organization and design of work;
6. Staff training and continuing professional development;
7. Establishing a regulatory framework;
8. Improving the efficiency of resource use.

The authors also identified other issues related to the technological, scientific, business, social and political challenges of an I4.0 company: development of intelligent devices, construction of network environment as cooperation between different systems, CPS modeling, integrations of CPS, testing of CPS, big data analytics, and digital manufacturing.

In [20], a systematic literature review was conducted to explore the challenges of Industry 4.0 based on the following principles: interoperability between humans and CPS, virtualization of CPS environment, real-time capability to react to any changes in the environment, decentralization of responsibility on the organizational hierarchy, service orientation architecture as software design principles, security and privacy of information using ICT technologies. 

The article [21] discusses the Industry 4.0 challenges and consequences from the workforce point of view, such as filling the employees’ knowledge gap, standardizing intelligent assistance systems, revising existing job profiles with more interdisciplinary skills, and re-organizing lifelong learning, which impacts work-life balance. 

Respectively in [22], the authors, through the review of the relevant literature and a survey conducted with 67 industries in Austria, list the industrial issues related to the demographic change that has taken place in recent years. At the same time, they suggest ways (based on Industry 4.0 technologies) to enable the elderly workforce to join and deliver as much as possible to the industry.

The authors of the article [23] define a conceptual framework that divides technologies into objectives for the companies: Smart Supply Chain, Smart Working, Smart Products, and Smart Manufacturing. The framework results from a survey performed on American companies and analyzed with statistical and clustering methods. The survey also derived the level of difficulties implementing Industry 4.0 technologies in big and small companies.

Finally, the article [24] aims to find the challenges facing five Swedish manufacturing companies in digitizing and automating their product and production platforms and explore ways to solve them. According to the interview results, the surveyed industries face problems related to simulation and calculation, automation, and data management. The first category is divided into two subcategories: computation and verification. The former concerns the automatic execution of calculations and the use of computational models to ensure the correct operation of the system, while the latter concerns software to verify product designs before the production process. Regarding the problems related to automation, design and production automation were distinguished. Finally, data management comprises data migration, data collection, Manufacturing Execution System improvement, etc.

Table 1 presents a summary of challenges identified in the literature. All the challenges were assigned to the following groups: challenges related to customer acquisition and contract planning (CACP), challenges related to manufacturing process preparation (MPP), challenges related to the manufacturing process (MP), and challenges related to manufacturing process monitoring and improvement (MPMI). In the next step, we tried to assign existing and available on the Industry 4.0 technologies market. The sources of information have not been cited to avoid suspicion of surreptitious advertising.

The information presented in Table 1 was taken into consideration in the process of the research development. However, by studying the relevant literature, besides extracting information for the questionnaire development, we identified its limitations and flaws. Precisely, the existing studies focus on individual countries, such as the UK [18], Brazil [23], Austria [22], and Sweden [24], as well as specific industrial challenges. On the contrary, the presented research work involves industries from all over the world while adopting a more holistic approach aiming to identify the problems at all stages of the production process.

## 3. Work Methodology

### 3.1. Steps of the Work

**Figure 1:**
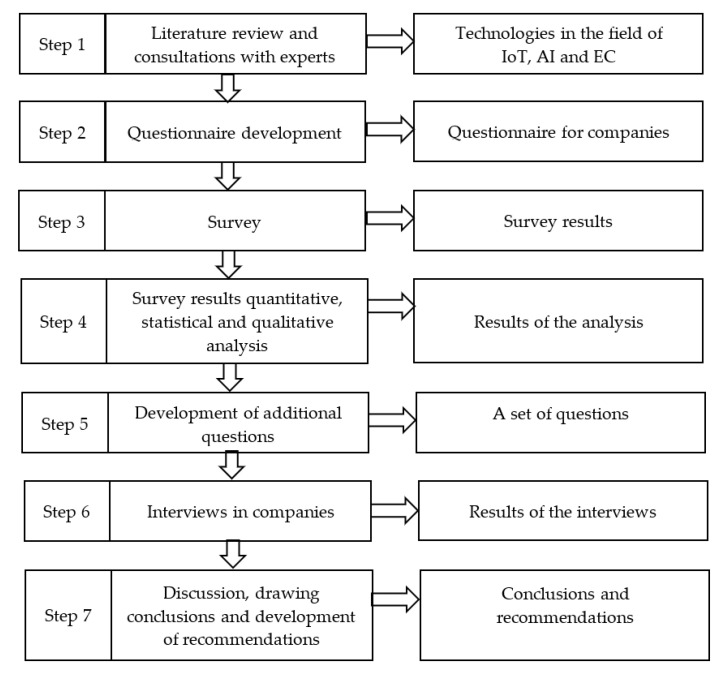
Work methodology.

Figure 1 presents the research methodology, consisting of seven steps. The first step was related to the preparation of the research, where a literature review (as presented in Section 2) and consultations with experts to develop survey questions were conducted. In the next step, a questionnaire was developed and shared with industrial companies. Then, the obtained answers were analyzed quantitatively and qualitatively. Based on the analysis results, a set of questions was developed and then used in interviews with industry representatives. Finally, based on the research results, conclusions were drawn regarding the needs of the industry, and recommendations were proposed.

### 3.2. Literature Review and Consultations with Experts

The performed literature review was insufficient to develop the survey questions on its own because, as shown in Table 1, there are some cells empty. The identified challenges did not cover all problems known to the authors from the experience of working closely with the industry. Besides, the internal firm perspective had to be considered in addition to the research community’s publications because many of them are theoretical and have little practical use. Therefore, consultations with experts were performed to prepare the survey questions. The purpose of consultations with experts was to evaluate selected topics regarding their importance for enterprises.

The second goal of the literature review was to identify Industry 4.0 solutions reported in publications as being available on the market and ready to be implemented in the industry. The literature review focused on areas where AI, IoT, and EC applications solve industrial problems.

### 3.3. Questionnaire Development

The questionnaire used in the research consists of:

- Multiple choice questions with predefined answers:
  o Yes; No; No, but we want to implement it—For business management and data flow systems;
  o Yes; No; Plan to have; Not relevant—For systems functionalities;
  o Important; Less important; Not relevant—For challenges;
  o Implemented in our company; We want to implement; Not relevant—For Industry 4.0 solutions.
- Open questions.
- Questions with predefined answers based on an extended Likert scale [25] with the following answers: not at all, to a small extent, to some extent, to a moderate extent, to a great extent, to a very great extent—For employees’ skills.

Moreover, the respondents had the option of adding additional comments.

The developed questionnaire was validated by asking a company to fill the questionnaire to ensure that the questions were clear for the respondents. The questions used in the survey are presented in what follows:

**General questions**

1. Company size
2. Industry
3. How many production machines do you have?
4. How many production sites do you have?
5. Production type
6. In which country (countries) the company operates?
7. In what department do you work?

**Detailed questions**

8 Do you use ERP system—Enterprise Resource Planning System?
9 What ERP system do you use?
10 Do you use MIS system—Management Information System?
11 What MIS system do you use?
12 Do you use MES system—Manufacturing Execution System?
13 What MES system do you use?
14 Do you use CRM system—Customer Relationship Management System?
15 What CRM system do you use?
16 Do you automatically transfer data between the internal systems?
17 Do you automatically transfer data from the system to supplier?
18 What type of data would you like to transfer automatically?
19 Does your client automatically transfer data to your system?
20 Do you automatically collect process data coming from different sensors in the system?
21 What are the data you automatically collect?
22 Do you have a system which…?
  - displays production orders from MRP/ERP
  - reports production order progress to MRP/ERP
  - automatically manages production orders
  - displays production jobs from MES
  - reports production jobs to MES
  - automatically creates production job for a specific process and equipment
  - indicates the equipment available to run a production job for a specific process
  - indicates the best equipment to efficiently run a production job for a specific process and product based on its job run history
  - displays material requirements for the production of a product
  - displays preparation steps for a specific process of a product
  - sends job information to the HMI (Human-Machine Interface) of the equipment
  - reports job production progress from equipment
  - stores a production recipe/technology for later use
  - sends a production recipe/technology to a compatible equipment with job information
  - displays product information from product management system (ERP, WebCenter…)
  - automatically manages product information, e.g., product change management
  - manages product related production process on equipment
  - displays related job and recipe/technology information for a specific product
23 Challenges connected with customer acquisition and contract planning
  - Business analysis
  - Relations with customers
  - Lack of consumers behavior prediction
  - Obtaining data from the market (competition, customers, potential customers, …)
  - Business process monitoring
  - Product design
  - Process design
  - Forecasting
  - Production/operations planning
  - Customer service
  - External logistics
24 Challenges connected with manufacturing process preparation
  - Planning of the materials/products deliveries
  - Purchasing process
  - Acquiring offers from potential suppliers
  - Negotiations with suppliers
  - Suppliers’ evaluation and ranking
  - Risk assessment of cooperation with suppliers
  - Comparing offers/suppliers
  - Purchase Price Variance (PPV)
  - Management of the materials/products deliveries
  - Warehouse management
  - Human resources management
25 Challenges connected with manufacturing process
  - Technology optimization
  - Product quality
  - Root cause analysis
  - Process stability
  - Machine operation monitoring
  - Tool management (e.g., drills, turning tools)
  - Unpredictable machine failures
  - Spare parts management
  - Maintenance personnel management
  - Forecasting of maintenance needs (health monitoring)
  - Planning the servicing of machines and devices (external service)
26 Challenges connected with manufacturing process monitoring and improvement
  - Production/operations process monitoring
  - Energy consumption management
  - Cost management
  - Internal logistics
  - Information flow
  - Data collection
  - Quality of the information
  - Access to information
  - Data analysis
  - Decision-making
  - Setting priorities of improvement actions
27 Other company challenges
28 What Industry 4.0 solutions are important for your company?
  - Intelligent tool (e.g., drills, turning tools) management system
  - Automated Guided Vehicles (AGV)
  - Decision support systems
  - Autonomous production systems with robots
  - Business intelligence
  - Predictive maintenance
  - Intelligent process diagnosis
  - Intelligent process supervision
  - Big data analytics
  - Automatic data collection
  - Supervisory control and data acquisition (SCADA) system
29 Other Industry 4.0 solutions implemented
30 Other Industry 4.0 solutions which we want to implement
31 To what extent your company has practical skills in the following technologies?
  - Artificial Intelligence
  - Machine Learning
  - Internet of Things
  - Edge Computing
  - Autonomous Systems
32 Which skills your company needs to deal with the current problems?

### 3.4. Survey

**Figure 2:**
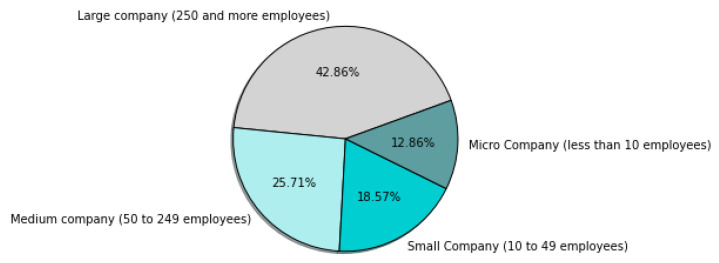
Percentage of companies based on company size.

**Figure 3:**
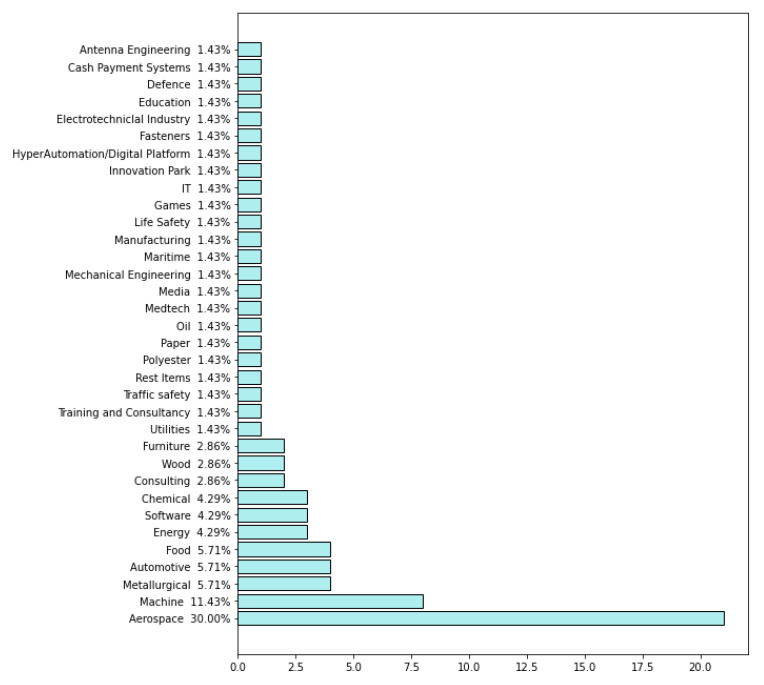
The percentage of questionnaires received from each industry.

**Figure 4:**
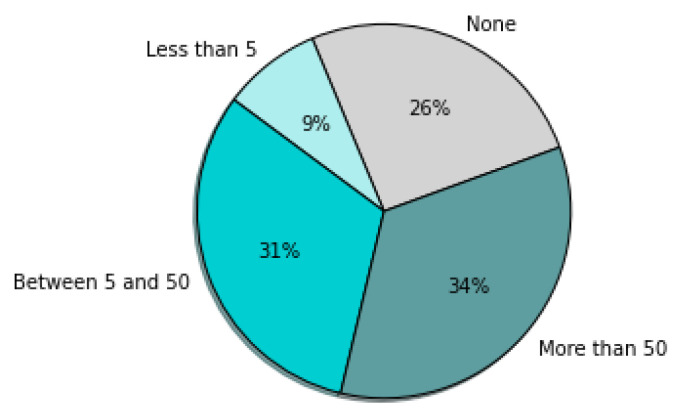
Number of machines in the analyzed companies.

**Figure 5:**
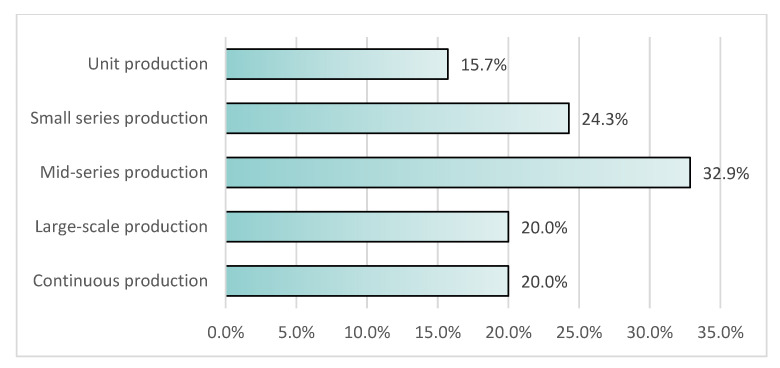
Percentage of questionnaires indicating the use of a specific type of production (one company can carry out several types of production).

**Figure 6:**
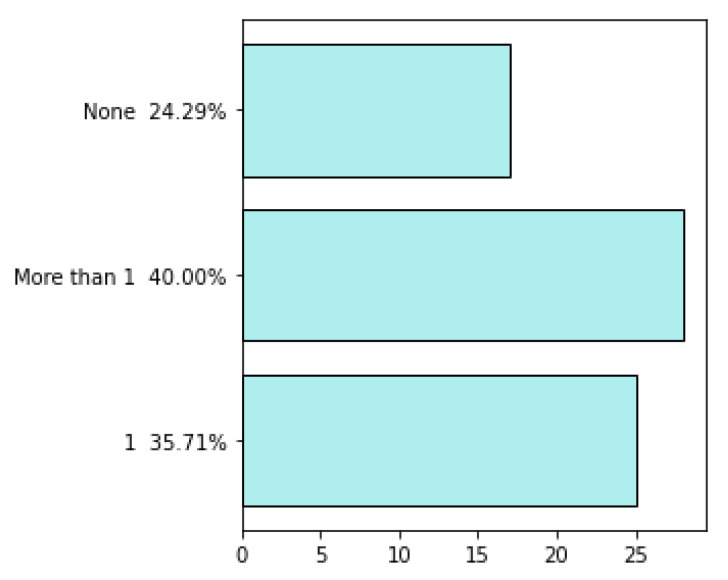
Number of production sites.

**Figure 7:**
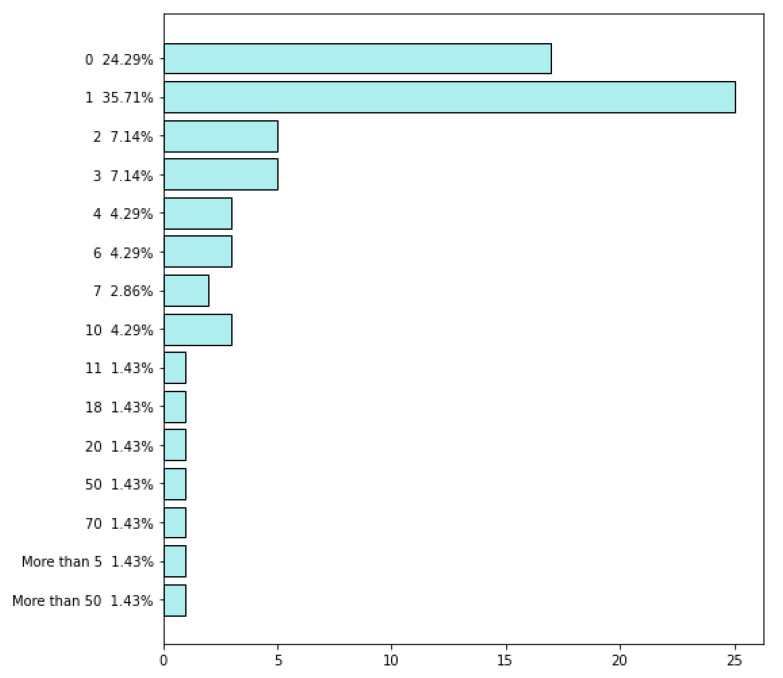
Number of production sites (details).

**Figure 8:**
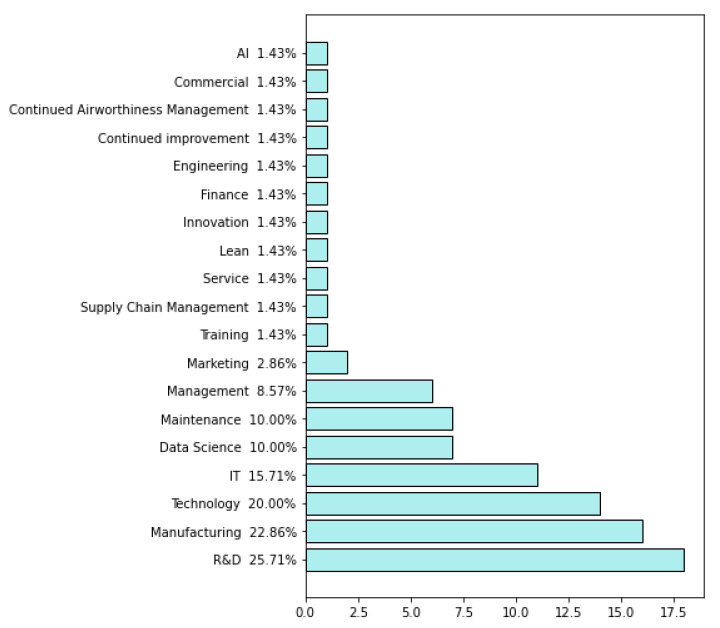
Departments represented by employees taking part in the research.

The survey was continued from 8 March 2021 to 12 April 2021, when the companies answered the questionnaire. As a result, 73 questionnaires were obtained and then evaluated in terms of their quality before they were analyzed. Finally, seventy questionnaires were further analyzed, while three questionnaires were excluded because information about countries where the companies operate was not presented. General information about the participating companies is presented in Figure 2, Figure 3, Figure 4, Figure 5, Figure 6, Figure 7 and Figure 8.

In this survey, most of the European countries were represented. The companies indicated that they operate in the following countries or regions: Poland (22 questionnaires), Spain (12 questionnaires), Italy (11 questionnaires), Germany (8 questionnaires), France (7 questionnaires), Greece (6 questionnaires), Portugal (5 questionnaires), Romania and United Kingdom (4 questionnaires from each), China, Russia, Slovakia and USA (3 questionnaires from each), Belgium, Canada, Croatia, Mexico, South Africa, Turkey, Ukraine (2 questionnaires from each), Australia, Austria, Brazil, Chile, Czech Republic, Egypt, Hungary, India, Ireland, Israel, Canada, Kazakhstan, Lithuania, Norway, Netherlands, Oman, Switzerland, United Arab Emirates, America, Asia, Australasia, EU, Europe and Scandinavia (1 questionnaire from each). One response stated that the company operates worldwide. The authors assumed that the number of countries is sufficient to draw safe conclusions. Moreover, the authors believe that the information from the represented departments’ employees is valuable in terms of the analyzed topics.

### 3.5. Survey Results Quantitative, Statistical and Qualitative Analysis

The survey results were subjected to quantitative, statistical, and qualitative analysis. The purpose of the quantitative analysis was to determine what percentage of the surveyed enterprises provided specific responses. 

Regarding the statistical analysis, the Chi-square test was performed for the chosen data to test the adopted hypotheses. The Chi-Sq test results are presented in the form of Pearson chi-square statistic (Chi-Sq), Degrees of Freedom (DF), and *p*-Value. A significance level of 0.05 was adopted. Therefore, when *p*-value ≤ 0.05, the variables are statistically significant, and the hypothesis is rejected. In the survey, for questions which have been subjected to the statistical analysis, the companies could indicate the following answers: (1) ‘Yes’, (2) ‘No’, (3) ‘No, but we want to implement’. While preparing data for the statistical analysis, we have created two data groups: with the answer ‘Yes’ and with the answer ‘No’ (including ‘No’ and ‘No, but we want to implement’). The reason for grouping the data was insufficient data in some categories for statistical analysis.

The qualitative analysis aimed to extract relevant information from the responses to open-ended questions.

### 3.6. Development of Additional Questions

The survey results were the basis for developing a list of additional questions used in the second phase of the research, namely in the interviews with company representatives. The list of questions is presented in the following:

1. How do you understand I4.0?
2. What possibilities for I4.0 implementation do you see in your company?
3. How do you understand IoT?
4. How can IoT support manufacturing and company management in your company?
5. How do you understand AI?
6. How can AI support manufacturing and organization management in your company?
7. How do you understand EC?
8. How can EC support manufacturing and organization management in your company?
9. What is your knowledge on condition monitoring and predictive maintenance?
10. How do you see the possibilities of condition monitoring and predictive maintenance implementation in your company?
11. What are your needs in the area of automated data collecting, analysis, and transfer between different internal IT systems in your company.
12. What are your needs in the area of automated data collecting, analysis, and transfer between IT systems used in your company and IT systems used by external actors in the value chain (i.e., suppliers, customers)?
13. What is your knowledge on ERP systems?
14. What are the purposes and areas of the ERP systems and what are the system’s main functionalities?
15. What are the barriers in your company in the implementation and effective use of ERP?
16. What is your knowledge on MIS systems?
17. What are the purposes and areas of the MIS systems and what are the system’s main functionalities?
18. What are the barriers in your company in the implementation and effective use of MIS?
19. What is your knowledge on MES systems?
20. What are the purposes and areas of the MES systems and what are the system’s functionalities?
21. What are the barriers in your company in the implementation and effective use of MES?
22. What is your knowledge on CRM systems?
23. What are the purposes and areas of the CRM systems and what are the systems functionalities?
24. What are the barriers in your company in the implementation and effective use of CRM?
25. What are the problems in building large-scale databases with different data from different sources?
26. What methods do you use for data analysis?
27. What is your experience in AI implementation in your professional career?
28. What is your experience in IoT implementation in your professional career?
29. What is your experience in EC implementation in your professional career?
30. What was the most difficult to understand and to implement AI?
31. What was the most difficult to understand and to implement IoT?
32. What was the most difficult to understand and to implement EC?
33. What are your current and future needs in knowledge and skills of your company employees to implement I4.0 solutions?

### 3.7. Interviews in Companies

Interviews were performed in the chosen companies by university employees researching to understand better the companies’ current and future needs. The discussion on the performed interviews is presented later in this work.

The goal was to reach up to 3 persons from the companies who were willing to be a part of the extended interview, so the company challenges could be presented from different perspectives. The interview was planned for a one-hour discussion. Since this interview aims to assess company needs, a group session with a few company employees could be organized.

Therefore, additional questions were prepared and asked the companies (see Section 3.6). This process lasted from 3 May 2021 to 7 May 2021.

### 3.8. Discussion

The discussion (Section 7) presented in this paper answers the following questions helping to conclude and prepare recommendations for companies that strive to digitize their processes and improve communication in their supply chain.

RQ1: What kind of systems have the companies implemented to improve the processes’ digitalization?

RQ2: What are the companies’ problems, and what technologies the companies implement or could implement to solve these problems?

RQ3: What are the industrial needs, and how can they be met to facilitate the digitization of enterprises?

## 4. Results Presentation and Analysis

### 4.1. General Overview

Section 4 of this paper presents and analyzes the data collected in the study. First, systems used in companies, particularly production companies, are presented. Next, data related to automatization in data transfer are shown, and then system functionalities are analyzed. Finally, companies’ challenges are summarized. 

### 4.2. Systems Used in Companies

**Figure 9:**
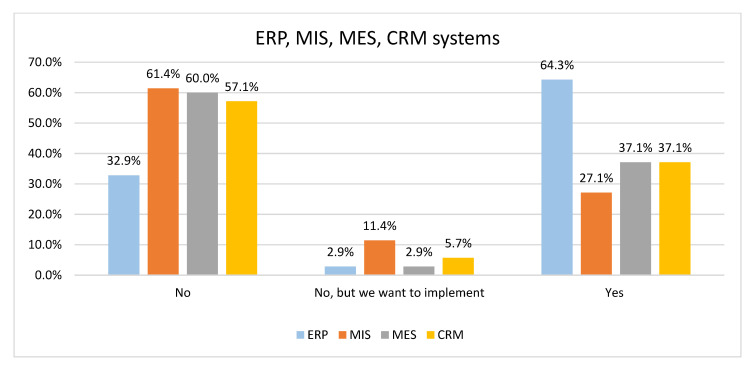
ERP, MIS, MES, CRM systems utilization in the studied companies.

This section outlines what systems the companies use and what systems they intend to use. From Figure 9, we see that 64.3% of companies have implemented an Enterprise Resource Planning (ERP) system, while 2.9% of companies want to implement such a system. More companies (11.4%) want to implement a Management Information System (MIS), 2.9% of companies want to implement a Manufacturing Execution System (MES) and 5.7% of companies want to implement a Customer Relationship Management (CRM) system.

**Figure 10:**
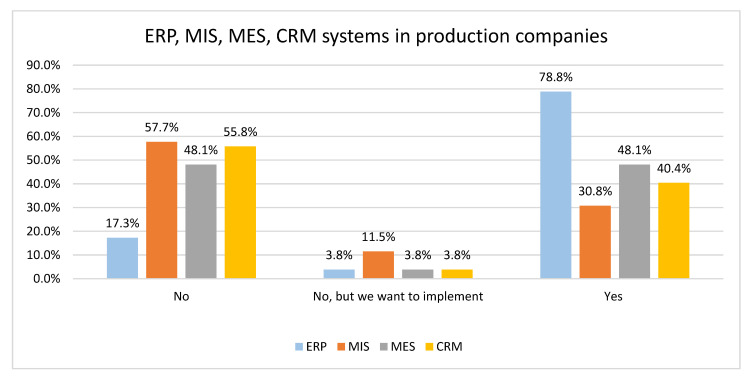
ERP, MIS, MES, CRM systems in the production companies.

Figure 10 focuses on systems utilization in production companies. It can be seen that 78.8% of production companies have an ERP system implemented, and only 17.3% of production companies have no ERP system.

To compare data obtained from production and non-production companies, we have performed the Chi-Square Test. Based on the results obtained, we have concluded that there is no statistically justified difference in the strategy of implementing MIS and CRM systems (*p*-value equals 0.486 and 0.392, respectively). However, there is a difference in the strategy of implementing ERP and MES systems (*p*-value equals 0.000). For example, 41 production companies declared that they have implemented an ERP system, and 25 have an MES system. In comparison, only 4 non-production companies have an ERP system, and one company has an MES system. 

It is interesting that a high percentage of companies do not have these systems and do not declare their future implementation.

**Figure 11:**
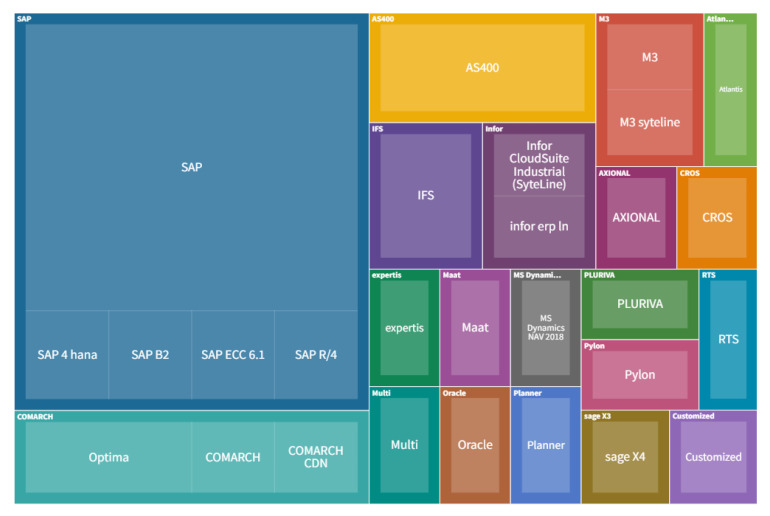
ERP systems are implemented in the companies.

**Figure 12:**
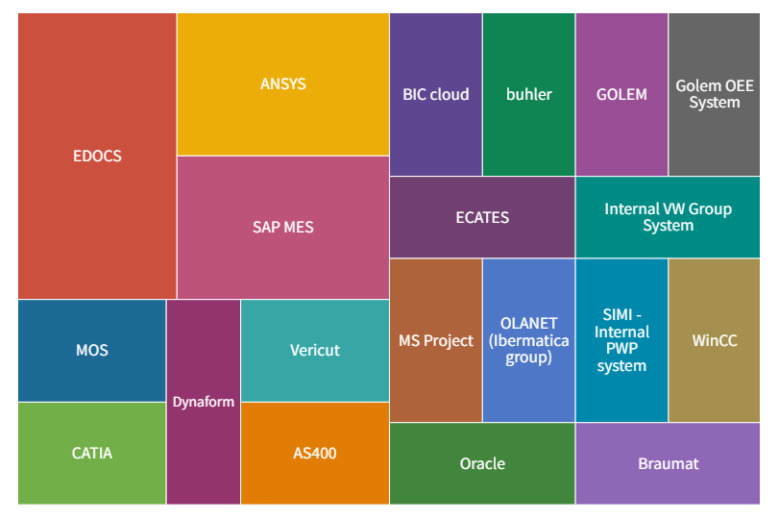
MES systems implemented in the companies.

**Figure 13:**
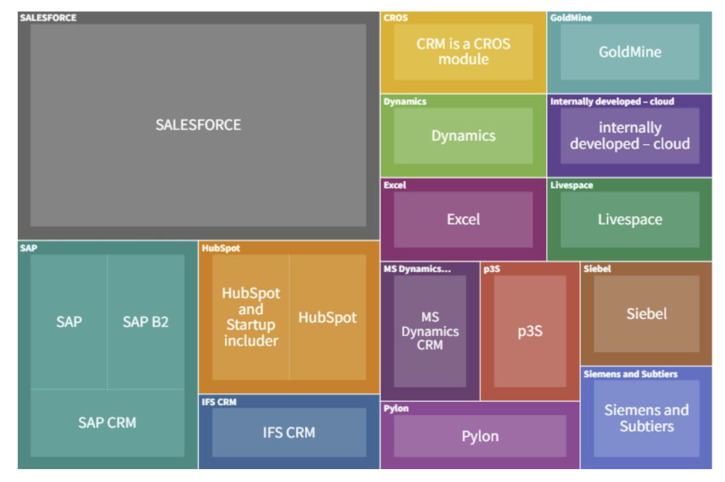
CRM systems implemented in the companies.

Figure 11 specifies the implemented ERP systems in the companies, Figure 12 shows MES systems, and Figure 13 shows CRM systems. Regarding the MIS systems, the companies have employed the following products: Asana, BCS, CIL, PowerBI, BI, Idk, ISO 27001, JIRA, Confluence, Multi, Oracle, Prisma 3, Qlik View, 1010Data and analytics built on AWS technology and various analytical tools like Tableau, Data Robot and other tools, Customized.

According to the above figures, the most frequently implemented ERP system is SAP. Companies implement various MIS systems, also customized. The most frequently indicated MES system is EDOCS. At the same time, the SALESFORCE system was indicated most often among all CRM systems used in the investigated companies.

Companies should be supported in expanding the knowledge of employees about the types and functionalities of IT systems which are of crucial importance for Industry 4.0, dedicated to supporting the companies’ functions. This is especially important about MIS systems, as most companies indicated that they plan to implement such systems.

### 4.3. Automatization in Data Transfer

**Figure 14:**
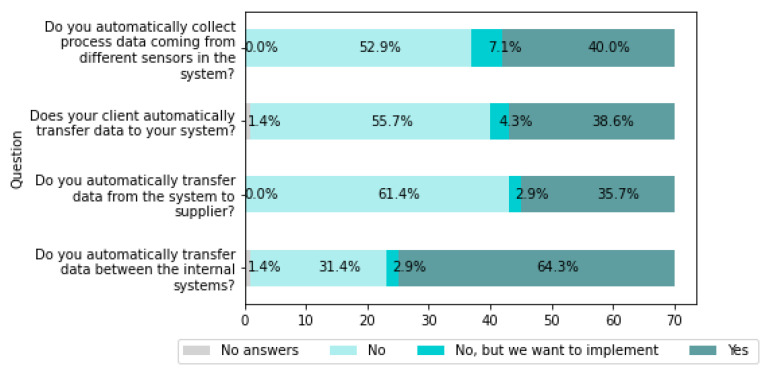
The functionalities of the systems related to the data transfer automatization implemented in the companies—number of questionnaires and percentage of answers in the total number of 70 questionnaires.

This section, presents data types that are automatically transferred and what companies want to improve in this area. Figure 14 summarizes the data.

Figure 14 shows that 2.9% of companies want to implement automatic data transfer between internal systems, and 64.3% of companies have already implemented this functionality. A total of 2.9% of companies want to implement automatic data transfer to suppliers, while 61.4% currently do not have this functionality. 

A total of 7.1% of companies want to collect process data using sensors automatically; 40% already do it. In 38.6% of companies, clients automatically transfer data to a company system.

Companies also answered that they want to transfer automatically:

- Material requirements and orders,
- Demand, planning data, quality requirements.

Companies indicated that they automatically collect the following data:

- Data coming from monitoring, sensor data, machine data, pressures, flows, process parameters,
- Production, machine and component parameters, machine tools monitoring, speed, quantity,
- Data from machine reports in production integrated with the ERP system; the data is used, among others, to control/monitor the use and efficiency of machines and the consumption of materials in the production process,
- Units produced and quality defects,
- Test results, measurement results,
- Product process data (SPC, Field, downtimes. etc.),
- Critical process data (temp, pH, conductivity, pressure), energy consumptions, production data (volumes, performance, losses, security),
- Environmental condition data, asset condition data,
- Current, voltage, uses, time, date, air temperature, humidity, machine status,
- Data from ships (fuel, speed, position, etc.),
- OEE (Overall Equipment Effectiveness),
- Surveillance radars, MLAT, ADSB,
- GPS coordinates and several time corrections,
- Metadata,
- Conversations.

The results show that companies are already using automated data transfer methods between internal systems to a relatively high degree. From this, it is concluded that companies’ current activities focus on integrating internal systems, which is the first step toward creating systems with automatic data transfer. The next step may be the integration of systems and automatic data exchange between different cooperating entities, i.e., customers and suppliers.

The results analysis concludes the need to support companies in the technical implementation of automated data exchange systems to speed up transformation to Industry 4.0, particularly communication between the various cooperating entities (suppliers, customers). Companies also need support in promoting knowledge among employees about the necessity and legitimacy of automated data transfer between systems, especially between different cooperating entities.

### 4.4. System Functions

**Figure 15:**
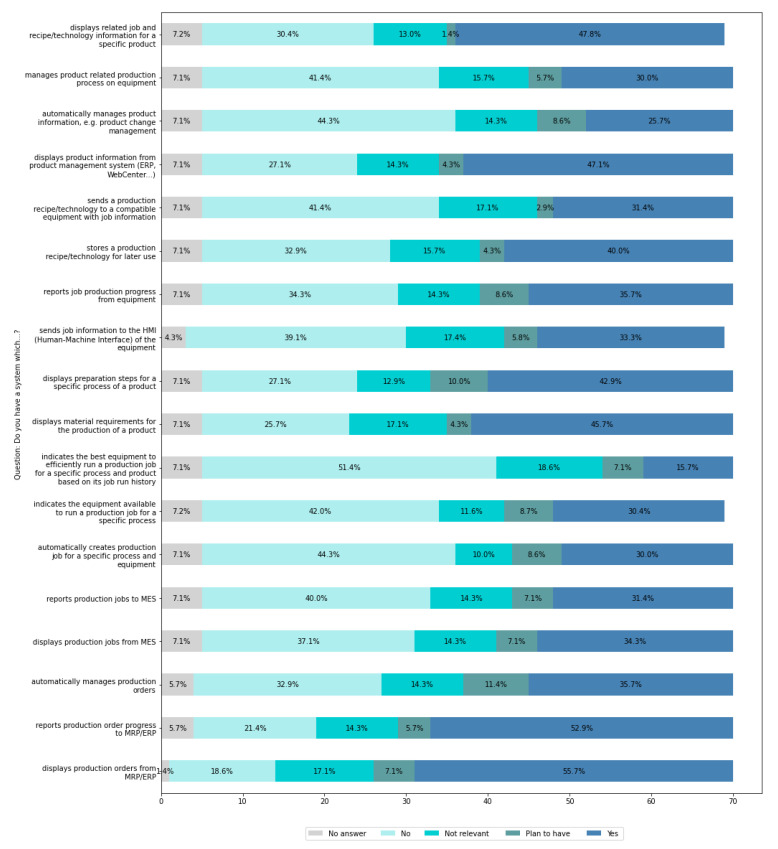
The functionalities of the systems implemented in the companies—number of questionnaires in a total number of 70 questionnaires.

Computer systems are implemented in enterprises to support their functionalities and thus facilitate and accelerate the performance of various tasks. Figure 15 presents which functionalities are supported by the systems and which of them are implemented in the companies.

From Figure 15, it can be seen that the highest percentage of the companies that have not implemented the mentioned systems aim to implement a system that automatically manages production orders (11.4%), while 10% plan to have a system that displays preparation steps for a specific production process. A total of 8.6% of companies want a system that indicates the available equipment to run a production job for a specific process, as well as a system that automatically creates production jobs for a specific industrial process or equipment, reports the production progress of the equipment, and automatically manages product information (e.g., product change management).

Therefore, the main needs of companies are connected with the implementation of the functionalities mentioned above.

The results suggest that most companies are in the first stage of implementing the basic functionalities of MRP/ERP systems in the production area, i.e., displaying data on production orders and reporting production progress. Relatively many companies have functionalities that allow access to technical and technological information about manufactured products. Far fewer companies have implemented MES-class systems with full functionality, including especially automatic data transmission to the shop-floor level, that is, to operator stations and machines. There is a lack of advanced functionalities, including in particular automatic process management and the collection and machine data analysis of historical data to support efficient production process management.

Based on the obtained results analysis, it should be concluded that it is necessary to support production companies in the integration of MRP/ERP and MES systems and support in the implementation of the advanced functionality of MES-class systems as the primary and necessary step for implementing Industry 4.0 solutions in industrial practice.

Companies also need support in promoting knowledge among employees on how to use artificial intelligence methods to increase the efficiency of production processes.

### 4.5. Company Challenges

This section presents information on the challenges faced by companies. The challenges concern four areas that are divided based on management functions (planning, organizing, implementing, monitoring):

- customer acquisition and contract planning,
- manufacturing process preparation,
- manufacturing process realization,
- manufacturing process monitoring and improvement.

**Figure 16:**
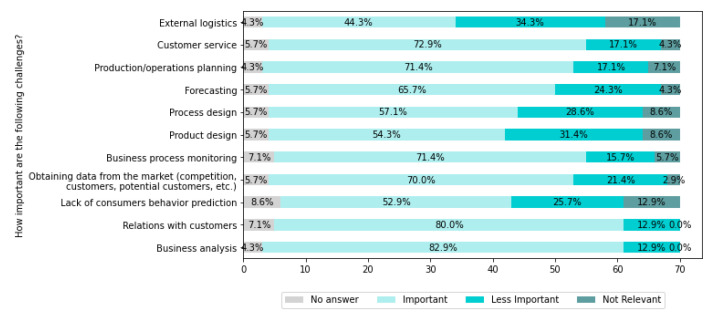
Challenges related to customer acquisition and contract planning—number of questionnaires in a total number of 70 questionnaires.

Figure 16 presents the challenges related to customer acquisition and contract planning.

From Figure 16, it can be seen that the most challenging are Business analysis (for 82.9% of companies), Relations with customers (for 80% of companies), Customer service (for 72.9% of companies), Business process monitoring (for 71.4% of companies), and Production/operations planning (for 71.4% of companies) in the area of customer acquisition and contract planning.

The main challenges identified above can be significantly supported by IT systems and technologies allowing automatic data collection from different sources and intelligent analysis using AI methods. Industry 4.0 technologies seem to meet the needs of today’s businesses. However, scientific research and systems development is required for the development of cognitive systems in order to meet the needs of businesses in the future entirely.

**Figure 17:**
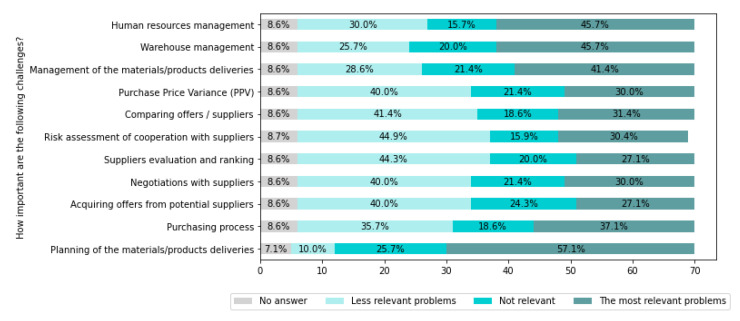
Challenges related to manufacturing process preparation—number of questionnaires in a total number of 70 questionnaires.

Figure 17 presents challenges related to the manufacturing process preparation.

From Figure 17, it can be seen that the most critical problems for companies are Planning of the materials/products deliveries (for 57.1% of companies), Warehouse management (for 45.7% of companies), Human resources management (for 45.7% of companies), Management of the materials/products deliveries (for 41.4% of companies), and Purchasing process (for 37.1% of companies).

IT systems can significantly support the challenges related to the preparation of production processes. Automatic data collection from various sources and their intelligent analysis using AI methods will support the optimization and planning of production processes.

The need for advanced support (negotiations, staff management) that can be achieved through cognitive methods and systems have also been highlighted.

**Figure 18:**
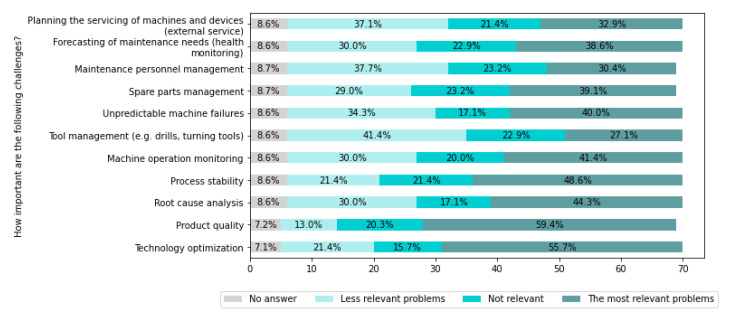
Challenges related to the manufacturing process—number of questionnaires in a total number of 70 questionnaires.

Figure 18 presents challenges related to the manufacturing process.

From Figure 18, it can be seen that the most important challenges are: Product quality (for 58.6% of companies), Technology optimization (for 55.7% of companies), and Process stability (for 48.6% of companies).

Addressing the main challenges identified in this area can be significantly supported by effective and intelligent methods of monitoring, diagnosing/predicting, and supervising processes and devices, and planning and optimization. However, further scientific research is required to develop intelligent systems for machines and processes condition monitoring and failure prediction.

**Figure 19:**
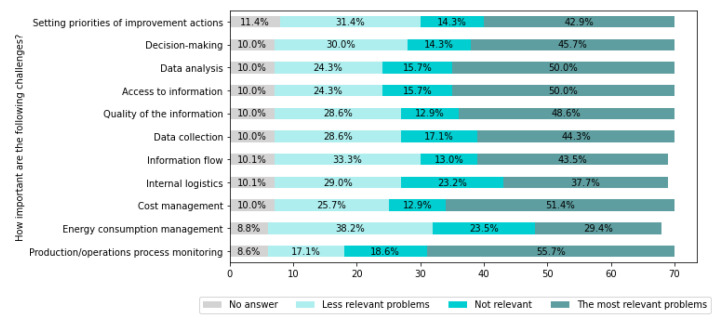
Challenges related to manufacturing process monitoring and improvement—number of questionnaires in a total number of 70 questionnaires.

Figure 19 presents challenges related to manufacturing process monitoring and improvement.

From Figure 19, it can be seen that the most important challenges are: Production/operations process monitoring (for 55.7% of companies), Cost management (for 51.4% of companies), Access to information (for 50% of companies), Data analysis (for 50% of companies), and Quality of the information (for 48.6% of companies).

Addressing the main challenges identified in this area can be significantly supported by advanced systems for data collection and intelligent analysis, i.e., MES systems integrated with machines, systems for condition monitoring, and failure prediction of machines and processes. 

However, advanced support (setting priorities, decision-making) requires the development of cognitive methods and systems. 

The companies also indicated other challenges, such as:

- Support maintenance line, associated logistics and spare parts control,
- Prediction of customers’ needs, btoc (business-to-consumer) and btob (business-to-business),
- Pricing,
- Challenges connected with corporates,
- Internal communication,
- Becoming an approved service center for a specific aircraft type,
- Greater demand the production,
- Industry 4.0 Solutions

In the aforementioned areas, companies require support for the transfer of knowledge about the possibilities of modern IT and AI systems and Industry 4.0 technologies, particularly their implementation into industrial practice. 

**Figure 20:**
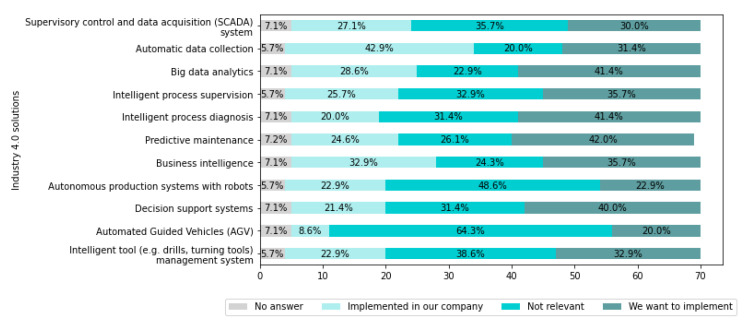
Important Industry 4.0 solutions for the companies—number of questionnaires in a total number of 70 questionnaires.

Figure 20 presents Industry 4.0 solutions which are important for the companies.

From Figure 20, it can be seen which technological solutions are the most desired to be implemented in the companies. These are Predictive maintenance (in 41.4% of companies), Intelligent process diagnosis (in 41.4% of companies), Big data analytics (in 41.4% of companies), and Decision support systems (in 40% of companies). Additionally, it is worth emphasizing that for 64.3% and 48.6% of companies, Automated Guided Vehicles (AGVs) for Autonomous production systems with robots, respectively, are not relevant.

Additionally, the companies indicated other Industry 4.0 solutions, which they have implemented, as follows:

- AI for behavioral analysis,
- Integration of machines for cutting upholstery fabrics with the ERP system (automatic sending of orders, reporting to ERP of order completion and the amount of material used),
- Virtual Assistants,
- AI Robots.

The companies also indicated other Industry 4.0 solutions which they want to implement, as follows:

- Digital Paperless Technology, PMI,
- Asset Condition Monitoring and Tracking,
- Automatic data analysis between machine tools/Coordinate-measuring machine (CMM)/other measuring devices—data correlation and data cross,
- Further automation of production processes along with integration with the ERP system.
- The use of machine learning algorithms for more efficient preparation and management of technological data,
- AI transport trolleys.

It was emphasized that the decision about which Industry 4.0 solutions to implement depends on the production line and area.

The main challenges identified above can be significantly supported on the operational level by existing IT systems and Industry 4.0 technologies. However, scientific research and systems development are required for the development of cognitive systems in order to fully meet the needs of businesses in the future on a tactical and strategic level.

**Figure 21:**
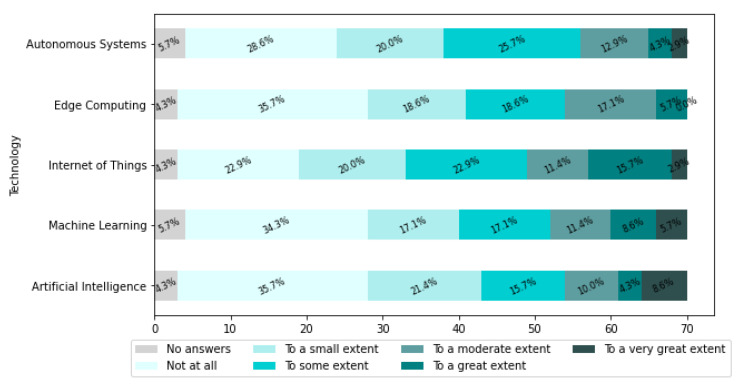
The extent to which the companies have practical skills in the presented technologies—number of questionnaires in a total number of 70 questionnaires.

Figure 21 presents the self-assessment results of companies’ competencies in AI, ML, IoT, EC, and autonomous systems.

Based on the information presented in Figure 21, it is noted that, in general, the majority of industries do not have the appropriate level of knowledge and skills to implement novel technologies, which is a prerequisite for the digital transformation of manufacturing systems. Therefore a significant problem facing industries is the lack of skilled labor.

Looking in detail in Figure 21, it can be seen that 8.6% of companies indicated that they have a high level of skills in AI. At the same time, 35.7% of companies admitted that they have no skills in AI. Also, 35.7% of companies admitted that they have no skills in EC. No companies indicated that they have a high level of skills in EC.

**Figure 22:**
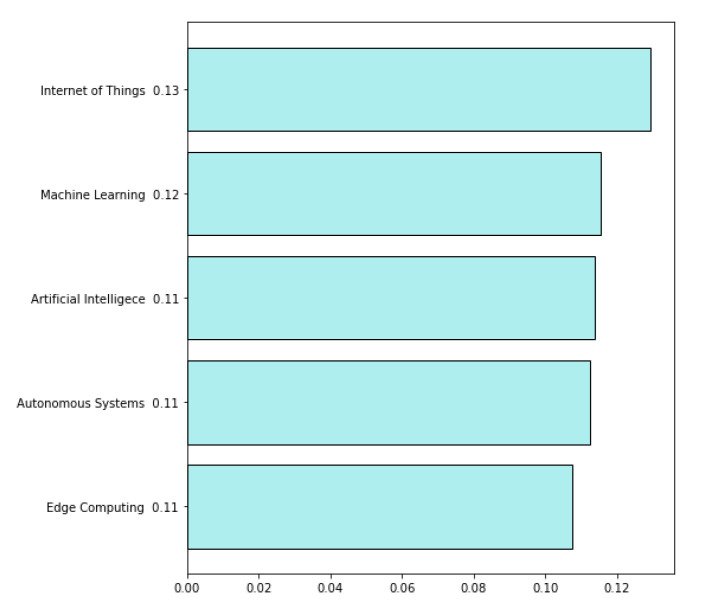
Practical skills level of the presented technologies (weighted average) in the range from 1 (not at all) to 6 (to a very great extent).

We have calculated the weighted average for each technology by translating the answers into numbers as follows: Not at all equals 1, To a small extent equals 2, To some extent equals 3, To a moderate extent equals 4, To a great extent equals 5, and To a very great extent equals 6, to assess which technologies are the least known in the companies. The level of skills calculated this way is presented in Figure 22. The most known is the Internet of Things and the least known is Edge Computing.

Additionally, the companies indicated the skills which they need to deal with the current problems, and they are:

- Programming, electronic hardware, and mechanical engineering, combined knowhow,
- Solving complex problems,
- Data science technology implementation, including AI to improve predictive maintenance,
- IT Development and Programming,
- skills required for professional profiles such as Data scientists, Digital architects, Full stack developers,
- Change management,
- Strategic Digital mindset,
- Designing solutions,
- Industrial automation skills, data security, IT,
- Artificial intelligence,
- Real Industrial IT—hardware and software,
- IoT knowledge to prepare functional diagrams of how systems should be connected (a lot of different equipment and systems),
- Greater openness on the part of top management.

Companies need support in developing competencies necessary for the effective implementation of Industry 4.0 solutions, both in the technical and soft areas, concerning current and future employees.

## 5. Statistical Analysis of the Results

### 5.1. Purpose of Statistical Analysis

The collected data were used in statistical analysis. We wanted to check whether:

- the size of the company is related to the fact that the company has an ERP, MIS, MES, or CRM systems implemented,
- the size of the company is related to the fact that the company has automatic data transfer implemented,
- type of production carried out in the company is related to the fact that the company has an ERP, MIS, MES, or CRM systems implemented,
- type of production carried out in the company is related to the fact that the company has automatic data transfer implemented,
- number of machines the company has is related to the fact that the company has an ERP, MIS, MES, or CRM systems implemented,
- number of machines the company has is related to the fact that the company has automatic data transfer implemented.

### 5.2. The Size of the Company and the Implemented ERP, MIS, MES, and CRM Systems

The following hypotheses were tested:

**Hypothesis** **1** **(H1).** *There is no difference in the number of companies that have implemented an ERP system regardless of the size of the company.*

**Hypothesis** **2** **(H2).** *There is no difference in the number of companies that have implemented an MIS system regardless of the size of the company.*

**Hypothesis** **3** **(H3).** *There is no difference in the number of companies that have implemented an MES system regardless of the size of the company.*

**Hypothesis** **4** **(H4).** *There is no difference in the number of companies that have implemented a CRM system regardless of the size of the company.*

The results of the Chi-Sq analysis are presented in Table 2.

**Figure 23:**
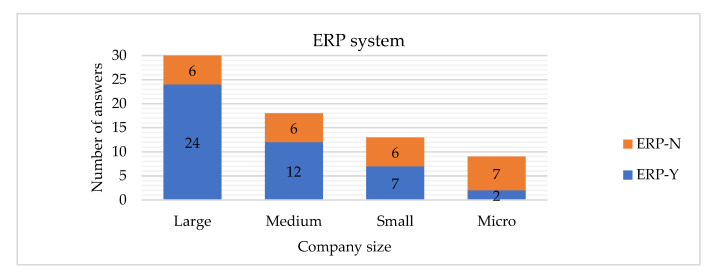
Number of companies having ERP system implemented (ERP-Y) or not (ERP-N) by company size.

The Hypothesis H1 is rejected, which means that size of the company is related to having implemented the ERP system. As shown in Figure 23, the larger company, the higher probability that the company has an ERP system implemented, which other studies have also verified [26].

### 5.3. The Size of the Company and the Implemented Automatic Data Transfer Systems

The following hypotheses were tested:

**Hypothesis** **5** **(H5).** *There is no difference in the number of companies that have implemented an automatic data transfer between the internal systems regardless of the size of the company.*

**Hypothesis** **6** **(H6).** *There is no difference in the number of companies that have implemented automatic data transfer from the system to the supplier, regardless of the size of the company.*

**Hypothesis** **7** **(H7).** *There is no difference in the number of companies that have implemented automatic data transfer from client to the system regardless of the size of the company.*

**Hypothesis** **8** **(H8).** *There is no difference in the number of companies that have implemented automatic data collection from sensors regardless of the size of the company.*

The results of the Chi-Sq analysis are presented in Table 3.

**Figure 24:**
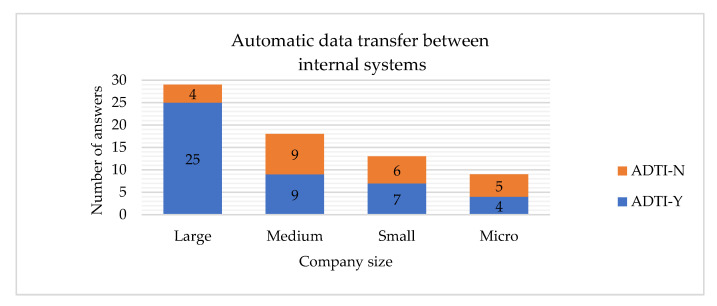
Number of companies having automatic data transfer between the internal systems implemented (ADTI-Y) or not (ADTI-N) by company size.

**Figure 25:**
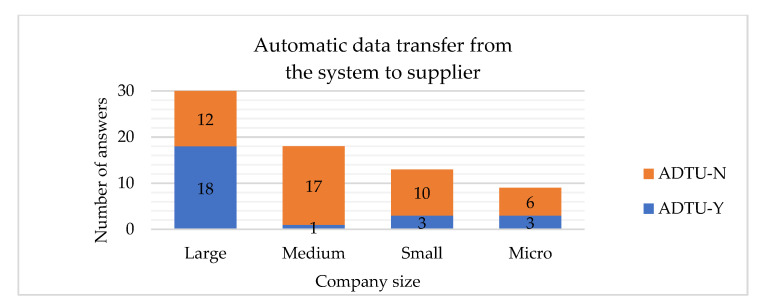
Number of companies having automatic data transfer from the system to supplier implemented (ADTU-Y) or not (ADTU-N) by company size.

The hypotheses H5 and H6 are rejected, which means that the company’s size is related to having the automatic data transfer between the internal systems and suppliers implemented. If the company is large, it is more probable to have such systems implemented (Figure 24 and Figure 25). Similar conclusions have been drawn by other researchers, stating that the company’s size is clearly related to the adoption of Big Data analysis systems [27].

### 5.4. The Production Type and the Implemented ERP, MIS, MES and CRM Systems

The following hypotheses were tested:

**Hypothesis** **9** **(H9).** *There is no difference in the number of companies that have implemented an ERP system regardless of the production type.*

**Hypothesis** **10** **(H10).** *There is no difference in the number of companies that have implemented an MIS system regardless of the production type.*

**Hypothesis** **11** **(H11).** *There is no difference in the number of companies that have implemented an MES system regardless of the production type.*

**Hypothesis** **12** **(H12).** *There is no difference in the number of companies that have implemented a CRM system regardless of the production type.*

The results of the Chi-Sq analysis are presented in Table 4.

Based on the statistical analysis results, we can say that the implemented systems are unrelated to the production type.

### 5.5. The Production Type and the Implemented Automatic Data Transfer Systems

The following hypotheses were tested:

**Hypothesis** **13** **(H13).** *There is no difference in the number of companies implementing an automatic data transfer between the internal systems regardless of the production type.*

**Hypothesis** **14** **(H14).** *There is no difference in the number of companies implementing an automatic data transfer from the system to supplier regardless of the production type.*

**Hypothesis** **15** **(H15).** *There is no difference in the number of companies implementing an automatic data transfer from client to the system regardless of the production type.*

**Hypothesis** **16** **(H16).** *There is no difference in the number of companies implementing an automatic data collection from sensors regardless of the production type.*

The results of the Chi-Sq analysis are presented in Table 5.

Based on the statistical analysis results, we can say that the implemented automatic data transfer systems are unrelated to the production type.

### 5.6. The Number of Machines and the Implemented ERP, MIS, MES and CRM Systems

The following hypotheses were tested:

**Hypothesis** **17** **(H17).** *There is no difference in the number of companies implementing an ERP system regardless of the number of machines.*

**Hypothesis** **18** **(H18).** *There is no difference in the number of companies implementing an MIS system regardless of the number of machines.*

**Hypothesis** **19** **(H19).** *There is no difference in the number of companies implementing an MES system regardless of the number of machines.*

**Hypothesis** **20** **(H20).** *There is no difference in the number of companies implementing a CRM system regardless of the number of machines.*

The results of the Chi-Sq analysis are presented in Table 6.

**Figure 26:**
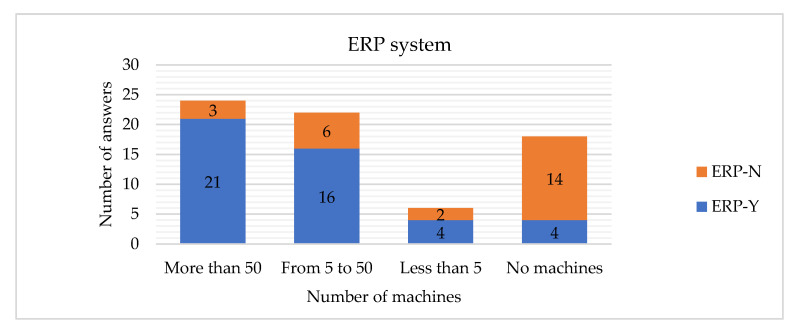
Number of companies having ERP system implemented (ERP-Y) or not (ERP-N) by number of machines.

**Figure 27:**
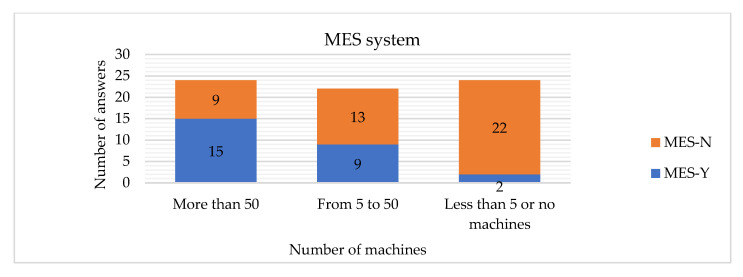
Number of companies having MES system implemented (MES-Y) or not (MES-N) by number of machines.

The hypothesis H17 is rejected, which means that the number of machines is related to the implementation of the ERP system. If the company has more than 50 machines, it is more probable that an ERP system will be implemented (Figure 26). If the company has more than 5 machines, it is more probable that an MES system will be implemented (Figure 27).

We expected that companies with fewer machines or without machines would not be willing to implement an MES system. Therefore, apart from the above-presented hypothesis H19, we have tested the following one: 

H19-1: There is no difference in the number of companies that have implemented an MES system regardless of the number of machines taking into account the companies which have (1) more than 50 machines, (2) from 5 to 50 machines, (3) less than 5 or no machines.

Testing the hypothesis H19-1, we discovered statistically justified differences between number of companies implementing MES systems if we look at the number of machines they possess (Chi-Sq = 6.993; DF = 2; *p*-Value = 0.030). Figure 20 shows that the more machines companies have, the more willingly they implement MES systems.

### 5.7. The Number of Machines and the Implemented Automatic Data Transfer Systems

The following hypotheses were tested:

**Hypothesis** **21** **(H21).** *There is no difference in the number of companies implementing an automatic data transfer between the internal systems regardless of the number of machines.*

**Hypothesis** **22** **(H22).** *Despite differences in the number of machines, there is no difference in the number of companies implementing an automatic data transfer between the system and the supplier.*

**Hypothesis** **23** **(H23).** *Despite differences in the number of machines, there is no difference in the number of companies that have implemented an automatic data transfer from client to the system.*

**Hypothesis** **24** **(H24).** *There is no difference in the number of companies that have implemented an automatic data collection from sensors regardless of the number of machines.*

The results of the Chi-Sq analysis are presented in Table 7.

**Figure 28:**
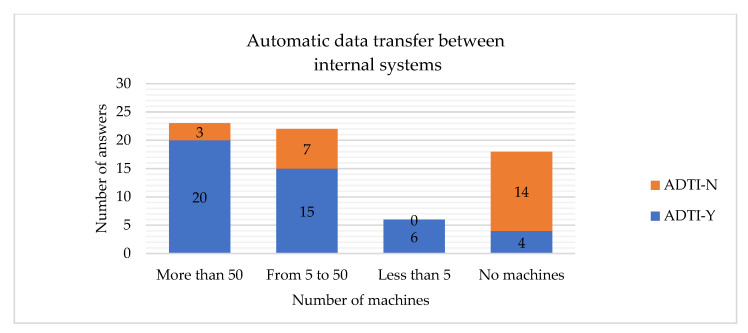
Number of companies having automatic data transfer between internal systems implemented (ADTI-Y) or not (ADTI-N) by number of machines.

**Figure 29:**
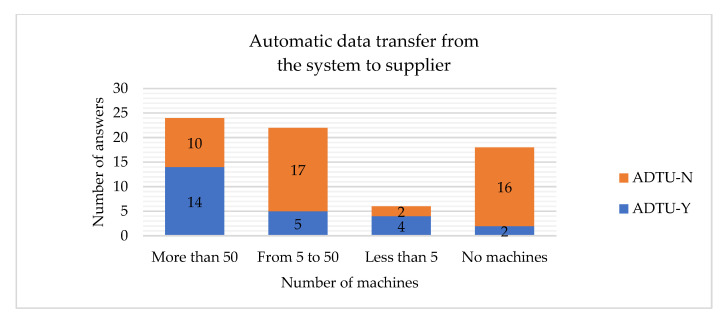
Number of companies having automatic data transfer from the system to supplier implemented (ADTI-Y) or not (ADTI-N) by number of machines.

The hypotheses H21 and H23 are rejected, which means that the number of machines is related to having the automatic data transfer between the internal systems and the client implemented. The larger company, the higher probability that the company will have such systems implemented (Figure 28 and Figure 29).

### 5.8. Summary of the Statistical Analysis

The statistical analysis was performed on the limited data set. Therefore, we can only draw conclusions about the surveyed companies.

Based on the analysis, we have drawn the following conclusions:

- The size of the company is related to the fact that the company has an ERP system implemented—the bigger the company, the higher probability that it has an ERP system implemented,
- The size of the company is related to the fact that it has implemented an automatic data transfer system between internal systems and suppliers—if the company is large, it is more probable that it has such systems,
- type of production carried out in the company is not related to the fact that the company has an ERP, MIS, MES, or CRM systems implemented,
- type of production carried out in the company is not related to the fact that the company has automatic data transfer implemented,
- The number of the company’s machines is related to the fact that it has an ERP and MES systems implemented—more machines mean a higher probability of having an ERP or MES system implemented,
- The number of the company’s machines is related to the fact that it has automatic data transfer between internal systems and with supplier implemented—the highest is that the company has such systems implemented if the number of machines is between 5 and 50.

## 6. Presentation of the Interviews Results

The interviews were performed in addition to the survey to better understand the companies’ problems and needs. The interviews were performed by the following university employees: Rzeszów University of Technology (PRZ), University of Pisa (UNIPI), Universitat Ramon Llull (URL), and University of Ioannina (UOI).

The needs identified during the additional interviews are presented in Appendix A and summarized in this section.

Researchers conducted interviews with employees of 20 companies. The main identified needs are as follows:

- Developing the competence of low-level staff to operate ICT systems. The companies’ employees need the training and courses to gain the ability to use ICT systems. Employees should be properly educated on the basics and have a good perception of the problems/tasks he/she undertakes. They should be able to find solutions on his/her own and should have the ability to lifelong self-learning.
- Understanding the purpose and idea of Industry 4.0 by mid-level staff. Employees of companies need training and courses to be aware of the opportunities offered by Industry 4.0 technologies for specific areas of business activity and increasing work efficiency. The employees should have the ability to identify areas where I4.0 solutions can be implemented and what solutions should be implemented. The employees should know what are the possible benefits of implementing I4.0 technologies, in particular economical savings—all expenses have to be justified. They should have the knowledge of the latest technologies together with embedded systems.
- Acquisition of practical skills in using AI tools and methods by senior-level staff. In addition to seminars, discussions, and expert panels that help raise knowledge, training and practical courses related to specific tools and software are needed.
- Developing the soft skills of employees involved in implementing ICT and Industry 4.0 in the company. It is essential to raise awareness of the need to implement Industry 4.0 in the company and build an active participation culture. In addition, employees should have logical thinking and teamwork skills, competencies in interpersonal communication, self-motivation, self-organization, self-management, ability to establish relationships, decision-making ability, and perseverance. Moreover, the ability for problem-solving and critical thinking are indeed major assets for an employee.

In particular, the companies indicated that it is essential to improve current professionals, teach them new technologies and their potential, and provide them with guidance on identifying the right technology for the right application scenarios for IoT. In work [28], the authors assess topics included in the university curricula that may facilitate the implementation of Industry 4.0 solutions. Moreover, the work [29] presents how future employees (students) assess their own competencies in this area.

Not only are employees with competencies in hardware and software, but also engineers with the competencies of “market adoption” are needed. The companies expect that their employees will be familiarized with the cloud platforms and be flexible to work at any platform seamlessly. The employees should also possess knowledge of programming and understand how this process works. They should have a general perception of the process, know to code, and be able to comply with a specific methodology. Finally, they should be trained in apprehending helpful information. Be familiar with extracting meaningful information from a vast amount of data. Therefore, developing employees’ competencies in data engineering and data science, e.g., creating automated dashboards and automated reports, is essential.

In data science, there is a need for standardization of data extraction, analysis, a broader view of data, from data collection to its application, and digitalization of the complete data gathering process. Getting the correct quantity and quality of data are crucial here. Thus, there is a need for data science and data management skills and more high-skilled analytics experts.

Moreover, support for human personnel by AI systems in data analysis and decision-making in current production management and technological processes is expected. The emphasis should be on quality assurance processes in to obtain high-quality outputs. Implementation of automatic quality control in different areas and automatic data acquisition is expected. Moreover, there is a need for data-driven cause-and-effect analysis. AI can also be used to predict a product performance during testing and the department’s end-of-month performance. AI systems can also help humans in detecting anomalies that need an intervention. Therefore, knowledge of AI capabilities and possible usage is needed.

Knowledge and possible applications of EC can help implement local data analysis, eliminating the need to transmit data to a central office. However, data security, powering and administration of EC entities, and retrofit of all these systems are required for their success. There is a need to secure the company’s network with modern technologies, as the company is vulnerable to cyber-attacks. Hard recovery after undergoing relevant company experiences in the past has proven the need for adopting and integrating advanced security systems in the company’s networks.

The companies also indicated the need to have a standardized state-of-the-art ERP system that can cater to the company’s needs that also tackle compatibility issues. The ERP systems should not be just logistics tools, but embrace AI technologies and provide a wide range of capabilities (not just financial management). Moreover, AI can support scheduling optimization. In general, there is a need to implement a system supporting the planning process.

In this regard, the reliability of the machines is critical. There is a need to monitor the condition of machines. Advanced predictive maintenance systems and systems for diagnosis and supervision of technological processes are required. For this, first of all, older machines have to be revamped to be able to collect data. Next, companies need to analyze the data captured from machines by monitoring different parameters necessary for compliance with current regulations. IoT should be implemented to keep machines running and monitor their operating parameters (e.g., vibrations) and parameters related to the working environment (e.g., emissions, humidity). The implementation of AI can improve production machines.

There is a need for I4.0 implementation for production by connecting all production components from all production sites in the cloud, using cross-data analysis to optimize production and logistics, and creating information for decision-making management. Effective introduction of MES is also needed. Moreover, implementing a centralized application/database for all the MES data (production data, production studies, quality data, etc.) is expected, such as consumption, waste management, production materials distribution, KPIs from PowerBI, etc. Implementation of aggregated statistics (up to now, they are segregated by markets with different CRMs) is also expected. Current solutions lack adaptability to companies’ internal processes. They focus on automation companies, mono-product, and mono-phase, in the case of different products and different phases. Therefore, significant changes have to be made (in terms of coding and for workers). At the same time, the workers feel under surveillance, and they do not like it. To overcome some workers’ fear of the Industry 4.0 revolution, robotics, etc., companies need to explain the advantages of I4.0. The second problem is replacing old-fashioned (or legacy) systems that cannot adapt to the new era because compatibility issues emerge between different APIs.

There is a need for employees with knowledge in data analytics, AI, and machine learning combined with business. Technologic solutions should be proposed based on the company’s needs/strategy. Studies should unify the business world with the software world and industry (agile), or fill the gap between them, while engineers should have more business knowledge. In addition, there is a need for people who could function as the liaison between the researcher on data analytics and the final product. They should understand both sides: the state-of-the-art in data analytics and the customer’s demands.

The companies are also interested in the implementation of such technologies as:

- Virtual Reality in the direction of conducting simulations useful for the company’s engineers to test their designs and drawings and training employees to perform various industrial tasks such as manual assembly, maintenance of industrial equipment, robotic arm programming, etc.
- Automated processes using robots.
- Robotic arms that bear intelligence and can function autonomously and be used for synchronization of production lines.
- Machine Learning for robots and product maintenance.
- Automated systems for machine retooling with intelligent decision support.

ICT and Industry 4.0 systems implementation processes have to be improved. Quality and efficiency by using standards and unified platforms for data collection and integration have to be ensured, while the implementation of data analytics is indispensable. Employees with programming and network skills and solid knowledge in automation for implementing Industry 4.0 are needed. Moreover, employees with strong competencies in electronics, antennas, and Radio Frequency, some knowledge of the wireless industry, radio protocols and their applications, English, and soft skills such as being a good listener, creative, etc., are required. 

The interviews also emphasized a need for employees’ competencies in Lean Manufacturing, production understanding, key performance indicators (e.g., OEE) calculation, and running projects using project management methods. Moreover, workers with more managerial skills, even at the cost of less specific expertise, are also indispensable.

Industry experts also mentioned the need for more collaboration with students in implementing IoT systems and I4.0 solutions on customer machines. However, neither students nor employers are sometimes ready to work with industrial IoT systems.

Among other needs, the companies indicated the following:

- The need for systems integration.
- Automated intralogistics systems.
- Development of I4.0 for product selling.
- Implementation of Business Intelligence for the Customers Journey.
- The need to overcome customers’ skepticism about data streams—edge data processing could help.
- Implementation of a more integrable antenna (plug & play) for IoT products.
- The mass-warning systems that incorporate sensors.

The interviews also underlined a demand for automation to share and transfer data with a customer.

## 7. Discussion

### 7.1. Systems Implemented in the Companies

This section discusses the first research question, which is RQ1: What kind of systems have the companies implemented to improve the digitalization of the processes?

Based on data collected in the research, it can be summarized that the companies have implemented different systems to carry out specific tasks or support specific processes carried out in enterprises, namely:

- Supervisory Control And Data Acquisition systems—SCADA,
- Enterprise Resource Planning—ERP,
- Manufacturing Execution System—MES,
- Project Management tools,
- Business Intelligence—BI,
- Management Information System—MIS,
- Databases—DBs,
- Customer Relationship Management—CRM,
- Computer-Aided Design—CAD,
- Data Analytics,
- Business Process Management—BPM,
- Information Security Management System—ISMS.

The listed systems support, among others, such enterprise functions as manufacturing and business process management, data management, security management, machines management, inventory management, infrastructure management, media management, and systems management.

According to the statistical analysis of the results, ERP and automatic data transfer systems are implemented by small, medium, and large companies. The larger companies are the ones that most commonly implement an ERP system. On the other hand, smaller companies (with less than 10 workers) do not implement ERP systems significantly.

Moreover, MES systems do not have a significant presence in companies without machinery or minimal productive machinery. These systems are more often implemented by companies that have more than 5 production machines.

### 7.2. Company Approaches for Problems Solving

In this section, the second research question is analyzed: RQ2: What are the companies’ problems, and what technologies do the companies implement or could implement to solve these problems?

Based on the research results, which included a literature review, information collection from different industries, and statistical analysis of them, the current companies’ problems as well as the technologies that implement or want to implement to solve these problems were identified. The need of industries for continuous and real-time supervision/monitoring of production processes which allows advanced decision-making and reduced reaction times for intervention, has made the connectivity and interoperability between industrial systems and devices a significant issue. Most companies still rely on their legacy systems, which consist of mechanical and analog devices, outdated Programmable Logic Controllers (PLCs), and obsolete industrial machines, since it is economically unmanageable and risky to replace all industrial equipment completely. However, due to the vendor-dependence nature as well as the limited processing and communication capabilities of the devices mentioned above, the interconnection between the industrial units and their connection to the cloud is a challenge. Therefore, a cost-effective and efficient way of integrating old appliances into modern production systems must be found. Advanced sensors and Industrial-Internet of Things (IIoT) gateways are an easy way. Precisely, smart sensors can measure different process parameters and send them to an IoT platform, while gateway devices with multi-protocol translation capabilities can collect and aggregate data from various I/O devices and controllers and communicate the data in local data centers or the cloud through IoT protocols.

Nevertheless, such an interconnected manufacturing ecosystem has acute problems regarding secure data sharing and cyberattacks in critical infrastructure networks. Data security has been repeatedly indicated as a challenge by the surveyed companies. It should be mentioned that partial or complete plant shutdowns, irreparable damage to essential devices, loss of products (e.g., changing the products’ information on shipping/labeling), and unauthorized data modification have been caused many times by such attacks [30]. Furthermore, through illegitimate access to production data, the design of a new product or the process recipe can be revealed, resulting in significant financial and reputational harm. In this regard, machine learning algorithms are employed to detect anomalous network traffic emanating from compromised IoT devices [31]. For the validation of these algorithms, well-structured datasets such as BoT-IoT [32], Edge-IIoTseT [33], and X-IIoTID [34] have been proposed. Therefore, IoT protocols that ensure data security and AI-based intrusion detection systems have been proposed [35,36,37,38,39]. Additionally, the edge computing paradigm could also be used since sensitive production data are processed closer to the source without being sent to the cloud.

An equally important issue is the unplanned production downtime which leads to productivity loss, possible equipment damage, or even accidents in the workplace. To prevent such errors, most industries, until now, follow periodic maintenance strategies that require system inspection and maintenance tasks to be performed at set time intervals, regardless of whether signs of machine performance degradation have occurred or not. The unnecessary equipment replacement and excessive use of maintenance materials such as lubricants and spare parts may increase economic waste. However, the advent of Industry 4.0 and predictive maintenance (PdM) can minimize the maintenance costs and the probability of production downtime. This is because following such a strategy, the industrial equipment maintenance is performed only when needed (just before the moment when it is likely to fail), thus allowing better management of maintenance materials. In addition, the Remaining Useful Life (RUL) prediction allows MRO inventory managers to effectively plan maintenance materials replenishment so that technicians can always deal with any problems that may arise. Additionally, according to 61.4% of participants, anomaly detection on an ongoing basis is also essential to improve the safety and reliability of the industrial system. However, for AI algorithms to be adopted in critical applications in the manufacturing domain, they must be characterized by interpretability beyond outstanding prediction accuracy. In other words, the model’s behavior should be understood at every step. The user can specify why this input data led to this prediction, when the model succeeds or fails, and when it is trusted. Thus, using explainable models in tasks such as fault detection makes it possible to find the root cause of failure. In more detail, 24.3% of the surveyed companies can predict future critical conditions, while 20% diagnose errors in real-time. For the implementation of predictive maintenance operations in the industrial environment, various solutions have been introduced in recent years, with the most significant percentage of them using AI methodologies such as machine learning, deep, learning or multiagent systems for automated decision-making, as well as IoT technologies for historical and present real-time data collection from sensors [40,41].

At the same time, the ever-increasing competitiveness between companies has turned product quality into another significant issue. Most companies have a quality inspector who inspects samples from the product batch during manufacturing. If the output does not meet the required standards, it is rejected while the process operator is informed to make the necessary adjustments to ensure quality. However, manual inspection has drawbacks, including a high probability of errors and the inability to continuously inspect the products’ quality. Through AI and intelligent cameras, systems that monitor the quality of each workpiece incessantly in real-time using process data with high levels of accuracy have been developed. After the valid data are initially collected (i.e., good and defective products’ images), the AI-powered software is trained in a supervised fashion to visually recognize specific classes of things (defective or non-defective). The model learns which product’s features are essential to define quality products during the training process, thus, creating a satisfactory system with precise, low error, and confident classifications [42]. As mentioned before, the manufacturing industry is one of the most dangerous workplaces, with more than 3000 occupational accidents and nine fatalities occurring each year [43]. Thus, apart from the production process’s efficiency and productivity optimization, the Industry 4.0 initiative aims to create a more sustainable industrial environment. Hardware advancement and IoT-based technologies have accelerated the development of Smart Personal Protection Equipment (PPE) solutions (e.g., smart helmets, clothes with wearable sensors) that monitor the environment (e.g., temperature, humidity, and atmospheric pressure). At the same time, AI-based software can detect possible risks inside the workplace.

Industries also face economic and environmental problems. IoT-based intelligent energy efficiency management systems (IoT-IEEMS) that monitor various process parameters such as temperature, humidity, energy consumption, and heating of different industrial devices have been utilized to solve this problem. In addition, a key technology underpinning Energy Optimization is AI. By utilizing machine learning algorithms combined with energy simulation software, industries can predict the system’s future behavior in terms of energy consumption, thus facilitating industrial process optimization [44]. 

Several companies also report inventory management problems, which have become more pronounced with the advent of COVID-19. On the one hand, product overages result in increased production costs for companies due to unused material or parts, while on the other hand, product shortages cause higher prices and delays. Therefore, machine learning models have been developed and used to predict production volume and consumer demand aiming to improve the following processes [45]:

- Better decision-making about the quantity of raw material to be ordered and whether new supply chains are needed or a reduction in the number of suppliers.
- Greater customer satisfaction
- Adaptation of marketing campaigns according to market preferences.
- Better planning of the production process based on the number of products that will probably be sold.

Nevertheless, to integrate IoT, AI, and EC in the industrial environment, employees must have the necessary practical digital skills and knowledge that many of them do not have. According to Deloitte and Manufacturing Institute, 2.1 million manufacturing positions will go unfulfilled by 2030 due to the digital skills gap [46]. One promising solution is adopting Virtual Reality (VR) and Augmented Reality (AR) technologies. Specifically, both VR and AR allow training using the “learning-by-doing” educational methodology where employees acquire the appropriate cognitive background and gain hands-on experience. Besides, these technologies can be assistive tools in several industrial operations such as product design, manual assembly, inspection activities, maintenance tasks, and order picking.

### 7.3. Company Needs

In this section, the third research question (RQ3) is analyzed: What are the industrial needs and how can they be met to facilitate the digitization of enterprises?

After conducting the aforementioned surveys and interviews with industrial companies, it can be concluded that there is a general lack of knowledge on how to implement the latest advances in the fields of AI, IoT, and EC to adopt the Industry 4.0 principles. More importantly, this lack of knowledge is not only identified on the technical side (i.e., importing a given technology inside the company’s ecosystem) but also on the awareness side (i.e., the company does not even know that such a technology exists or what can be used for). This fact became especially relevant when conducting the interviews with companies since most of the respondents asked for further clarification on the meaning of technical topics such as distributed computing, machine learning, etc. This subsection collects and summarizes the identified companies’ needs in the field of AI, IoT, and EC during the interview process and envisages a first step toward their addressing.

According to the insights obtained in this research, companies’ needs (compared with [23]) can be articulated into three dimensions: (1) data management, (2) knowledge transfer, and (3) training.

Data management refers to the challenges of processing the massive amount of data that modern industrial processes generate, ranging from data collection to data exploitation, including automation. When considering automatization in data transfer, it has been identified that companies need support in implementing integrated IT systems, including MES and MIS-class systems and their advanced functionalities. Specifically, it has been found that companies need to automatically transfer data from material requirements and orders as well as data from Demand, Planning, and Quality requirements. Also, these advanced functionalities of MES and MIS systems could assist companies in automatizing the data collection procedures in industrial environments, which has also been identified as a latent need in companies. Also, once data are somehow acquired by companies, it has been identified that they need support in the implementation of intelligent condition monitoring systems, especially with the functionality of failures prediction of processes and machines. Indeed, predictive maintenance has emerged as a hot research topic in the machine learning community in the last 5 years [47,48]. It is challenging to train a machine learning model robustly and reliably with inadequate (i.e., devices recently deployed) or highly imbalanced (i.e., the number of data instances belonging to the “normal operation” class is larger than the number of instances belonging to the “malfunction” class) historical data [49].

Knowledge transfer refers to the process of sharing (i.e., importing and exporting) experiences from and to others to enrich the whole industrial ecosystem. Specifically, it has been identified that companies need support in spreading the knowledge of the possibilities and practical methods of implementing integrated platforms for automatic data exchange between IT systems, including cooperating companies (i.e., suppliers and clients). Also, it has been identified that companies need support for knowledge transfer on the capabilities of today’s IT systems, AI, and other Industry 4.0 technologies, regarding their implementation into industrial practice on the operational level. Therefore, there is an apparent lack of an open platform or standard procedure for industrial companies to describe their (good) practices when using a given technology. Surprisingly, on the technological side, there are plenty of public repositories and open data platforms that support the idea of knowledge transfer between developers [50,51,52]. Maybe, this could be a good starting point to address these needs.

Finally, the training addresses the knowledge gaps that employers in companies currently have, and closing them will prepare companies to adopt new AI, IoT or EC technologies. Indeed, Industry 4.0 forces companies to include new job profiles (e.g., data scientists, data architects, data analysts, data engineers) in current industrial working environments [53]. As most of these new profiles did not exist in the past, companies often struggle to figure out how to integrate them into the team and which are the most appropriate tasks to assign them. Also, companies find it challenging to reskill current workers due to the high amount of content associated with AI, IoT, and EC for Industry 4.0 that are currently available. We have identified that companies need support in developing training materials for current and future employees to give them the hard (i.e., technical) and soft competencies required for the effective implementation of Industry 4.0 solutions. Therefore, blending Industry 4.0 specialists with industry professionals in a single training program might be a good approach to address this need. In this way, both profiles would understand each other language and would learn how to complement their skillset effectively to make Industry 4.0 a reality in the real world.

## 8. Conclusions

### 8.1. General Conclusions

In this paper, a questionnaire-based survey was conducted to investigate the used data/business management systems from industrial companies, the challenges they face nowadays and the technologies that implement (or could implement) to solve them, and finally, their needs regarding the digitization of the manufacturing ecosystem. Initially, with the review of the relevant literature, the survey questions asked in the industries were identified. Then, after analyzing the participants’ answers, interviews were conducted with companies’ representatives to determine their real needs. The results show that the highest percentage of industries is still in an early stage of their digitization since they have not invested yet in technologies like MIS, CRM, and MES software to gain efficiencies in critical business processes. At the same time, there is still enough room to improve the production process efficiency since more than 50% of enterprises have not implemented data exchange mechanisms between heterogeneous sources such as manufacturing systems, suppliers, and customers. The most critical issue is the lack of automatic machine data collection, which is the basis for intelligent manufacturing. In addition, the industrial problems can be addressed either by collecting data automatically from the shop floor using IIoT technologies (to monitor process parameters in real-time) or by implementing AI-based methods for quality control, anomaly detection, and predictive maintenance (i.e., condition monitoring, RUL prediction etc.) [54]. Nevertheless, while industries are aware of the advantages of implementing AI, IoT/IIoT, and EC to solve their problems, many industrial employees do not have the appropriate skills to integrate them. As a result, industry professionals’ current level of knowledge and skills in AI, ML, IoT, and EC was determined, leading to the conclusion that most industries lack the necessary competencies to implement novel technologies, which is a prerequisite for the digital transformation of manufacturing systems. 

Empirical data collected in this work suggest that reskilling/upskilling industry employers would have a significant impact on addressing the problems associated with the early adoption of digital technologies (e.g., MIS, CRM, MES), which would contribute to improving the production process efficiency. This reskilling/upskilling process could be conducted by combining some of (or all) the following activities:

1. Implementing an Employee Training and Development process with external teachers/researchers/domain experts by means of e-learning platforms. The flexibility provided by these platforms makes them very suitable in an industry context. Alternatively, it would be also possible to bring technology experts to industrial companies to identify and fill the knowledge gaps in a particular field.
2. Fostering collaborations with academia. That is, promoting the mentoring of students’ thesis or partnering with universities in technology transfer projects.
3. Using VR and AR solutions (see Section 7.2) to rapidly provide industry workers with valuable hands-on experiences to gain the necessary knowledge.
4. Enriching the training of young engineers from the stage of higher education through a modern curriculum by exposing them to real-world (i.e., industry) use-cases and up-to-date technologies. These use cases could include some tasks that consisted of working side by side with industry employers. This would be aimed to strengthen the link between universities (who train useful employees for the industry) and industry (who push the technological boundaries in our society).

From an educational perspective—and according to the collected insights—these reskilling/upskilling processes should mainly focused on the following topics:

- In the IoT/IIoT domain: Acquisition of knowledge related to the operation and monitoring of modern automation and intralogistics systems.
- In the AI domain: Acquisition of practical skills and competencies in data engineering, data science, AI tools, and data analytics for data analysis (e.g., predictive maintenance, systems diagnosis) and decision making.
- In the EC domain: Implementation of data processing on the edge to increase data security and reduce latency.

Additionally, a latent need regarding training in soft skills (e.g., involvement, logical thinking, teamwork, interpersonal and interdisciplinary communication, self-motivation, and self-organization) was identified as well. 

### 8.2. Work Limitations

However, as in any such study, we encountered different work limitations. The initial goal was to conduct large-scale research involving many industries worldwide. Unfortunately, industrial companies seem to be reluctant to participate since only 73 completed the questionnaire. Nonetheless, we believe that the collected data is sufficient to answer the defined research questions reliably. 

At the same time, one can see that the geographical distribution of industrial companies is not uniform. Knowing that the degree of digitization in industries differs significantly across countries, one can hastily conclude that the results are biased. However, through their analysis, similar trends were observed between companies from different countries, which endorses the reliability of the study. 

Another concern relates to companies’ replies on their level of knowledge and skills in AI, ML, IoT, and EC since the self-assessment via a scale may sometimes involve subjectivity. Nevertheless, according to the answers of each industry, it was concluded that they generally have the same cognitive background in the same technologies. We, therefore, consider that the answers largely reflect the current status of the industrial environment concerning the employees‘ digital skills. 

Finally, as previously pointed out, this research work is limited to presenting the industry’s current picture. However, it is not certain that the same conditions will exist in the future. The continuous evolution of AI, IoT, and EC implies a constant change in the manufacturing ecosystem.

### 8.3. Future Research

The presented article can serve as a guide for Industry 4.0 solution providers and researchers while assisting universities in creating a modern curriculum that could meet real industrial needs. However, both industry and academia need to have an even clearer picture of what is feasible and what needs to happen so that the digital transformation will occur. The implementation of new technologies should be based on a good strategy, depending on the company’s readiness for digitization [55]. To support the industry, we aim to develop a taxonomy concerning the enabling technologies in Industry 4.0 world and Industry 4.0 problems in a future study. Developing such a taxonomy would be a quick first step toward the successful implementation of Industry 4.0. Specifically, this taxonomy could (1) enable industrial companies to describe their problems without having in-depth knowledge regarding AI, IoT, and EC, (2) list existing I4.0 technologies presented in real-world use-cases, and (3) set up the fundamentals of a common language to be shared between Industry 4.0 practitioners and academics. In this way, this taxonomy would provide a standard means to formalize the description of the companies’ needs regarding Industry 4.0 problems that can be solved using technologies such as AI, IoT, and EC. This tool would help industrial companies better understand how available technologies could help them meet their needs while helping researchers identify the application areas of their developing technological solutions and practically contribute to the Industry 4.0 vision. 

## Figures and Tables

**Table 1:** Company Challenges from articles of literature review; CACP—customer acquisition and contract planning, MPP—manufacturing process preparation, MP—manufacturing process, MPMI—manufacturing process monitoring and improvement.

Company’s Challenges	The Group to Which a Challenge Has Been Assigned	Citations	Industry 4.0 Technologies
Implementation Costs	CACP	[18,20,21]	-
Implementation Time	CACP, MPP	[18,19,20,21]	-
Technology Knowledge	CACP, MPP	[18,19,20,21]	Business intelligence
Safety and security	CACP, MP	[19,21]	Smart Personal Protection Equipment Cybersecurity protocols
Establishment of a comprehensive and reliable industrial broadband infrastructure	MPMI	[19,20]	-
Horizontal and Vertical Integration	CACP, MP	[18,19,20,21,23]	Decision support systems
Traceability	MP, MPMI	[21,23]	Supervisory control and data acquisition (SCADA) system Automated Guided Vehicles (AGV)
Reduction of Energy Consumptions	MP, MPMI	[18,23]	Intelligent process supervision
Additive Manufacturing Implementation	MP	[18,20]	Additive Manufacturing
Predictive Maintenance	MPMI	[18,23]	Predictive maintenance
Simulation Implementation	MPP	[18,20,24]	Intelligent process diagnosis Intelligent tool (e.g., drills, turning tools) management system
Automated and Integrated Robots	MP, MPMI	[18,20,21]	Automatic data collection Autonomous production systems with robots
Big Data Analytics	MPMI	[18,19,20,21]	Big data analytics
Demographic Challenge	MP	[22]	Supporting technologies and assistance systems
Calculation Challenges	MPMI	[24]	Artificial Intelligence techniques
Data Management	MPMI	[24]	Product Lifecycle Management, CAD/CAM tools
Design Automation	CACP	[24]	IoT
Production Automation	MP	[24]	Machine Learning, Knowledge-Based Engineering, Digital technologies embedded in robots, IoT

**Table 2:** Results of Chi-Sq analysis for ERP, MIS, MES, and CRM systems depending on company size (Large, Medium, Small, Micro).

System	Hypothesis	Results of Chi-Sq Analysis
ERP	H1	Chi-Sq = 10.824; DF = 3; *p*-Value = 0.013
MIS	H2	Chi-Sq = 2.058; DF = 3; *p*-Value = 0.560
MES	H3	Chi-Sq = 5.074; DF = 3; *p*-Value = 0.166
CRM	H4	Chi-Sq = 0.996; DF = 3; *p*-Value = 0.802

**Table 3:** Results of Chi-Sq analysis for automatic data transfer depending on company size.

Automatic Data Transfer…	Hypothesis	Results of Chi-Sq Analysis
between the internal systems	H5	Chi-Sq = 9.923; DF = 3; *p*-Value = 0.019
from the system to supplier	H6	Chi-Sq = 15.764; DF = 3; *p*-Value = 0.001
from client to the system	H7	Chi-Sq = 6.183; DF = 3; *p*-Value = 0.103
Automatic data collection from sensors	H8	Chi-Sq = 2.977; DF = 3; *p*-Value = 0.395

**Table 4:** Results of Chi-Sq analysis for ERP, MIS, MES, and CRM systems depending on production type (Continuous production, Large-scale production, Mid-series production, Small series production, Unit production).

System	Hypothesis	Results of Chi-Sq Analysis
ERP	H9	Chi-Sq = 6.259; DF = 4; *p*-Value = 0.181
MIS	H10	Chi-Sq = 4.755; DF = 4; *p*-Value = 0.313
MES	H11	Chi-Sq = 7.041; DF = 4; *p*-Value = 0.134
CRM	H12	Chi-Sq = 1.913; DF = 4; *p*-Value = 0.752

**Table 5:** Results of Chi-Sq analysis for automatic data transfer depending on production type.

Automatic Data Transfer…	Hypothesis	Results of Chi-Sq Analysis
between the internal systems	H13	Chi-Sq = 3.740; DF = 4; *p*-Value = 0.442
from the system to supplier	H14	Chi-Sq = 3.321; DF = 4; *p*-Value = 0.512
from client to the system	H15	Chi-Sq = 2.271; DF = 4; *p*-Value = 0.686
Automatic data collection from sensors	H16	Chi-Sq = 0.714; DF = 4; *p*-Value = 0.950

**Table 6:** Results of Chi-Sq analysis for ERP, MIS, MES, and CRM systems depending on number of machines (More than 50, From 5 to 50, Less than 5, No machines).

System	Hypothesis	Results of Chi-Sq Analysis
ERP	H17	Chi-Sq = 20.203; DF = 3; *p*-Value = 0.000
MIS	H18	Chi-Sq = 2.634; DF = 3; *p*-Value = 0.452
MES	H19	Chi-Sq = 7.277; DF = 3; *p*-Value = 0.064
CRM	H20	Chi-Sq = 6.574; DF = 3; *p*-Value = 0.087

**Table 7:** Results of Chi-Sq analysis for data transfers depending on number of machines.

Automatic Data Transfer…	Hypothesis	Results of Chi-Sq Analysis
between the internal systems	H21	Chi-Sq = 22.745; DF = 3; *p*-Value = 0.000
from the system to supplier	H22	Chi-Sq = 14.214; DF = 3; *p*-Value = 0.003
from client to the system	H23	Chi-Sq = 6.923; DF = 3; *p*-Value = 0.074
Automatic data collection from sensors	H24	Chi-Sq = 4.749; DF = 3; *p*-Value = 0.191

## Data Availability

Not applicable.

## Abbreviations

ADTI	Automatic Data Transfer between the Internal systems
ADTU	Automatic Data Transfer from the system to supplier
AGVs	Automated Guided Vehicles
AI	Artificial Intelligence
AR	Augmented Reality
BI	Business Intelligence
BPM	Business Process Management
CACP	Customer Acquisition and Contract Planning
CAD	Computer-Aided Design
Chi-Sq	Pearson chi-square statistic
CMM	Coordinate-measuring machine
CPS	Cyber-Physical Systems
CRM	Customer Relationship Management
DB	Database
DF	Degrees of Freedom
EC	Edge Computing
ERP	Enterprise Resource Planning
I/O	Input-Output
I4.0	Industry 4.0
ICTs	Information and Communication Technologies
IEEMS	Intelligent Energy Efficiency Management System
IIoT	Industrial-Internet of Things
IoT	Internet of Things
ISMS	Information Security Management System
IT	Information Technology
M2M	Machine-to-Machine
MES	Manufacturing Execution System
MIS	Management Information System
MNE	Multi-National Enterprise
MP	Manufacturing Process
MPMI	Manufacturing Process Monitoring and Improvement
MPP	Manufacturing Process Preparation
MRO	Maintenance, Repair and Operations
OEE	Overall Equipment Effectiveness
PdM	Predictive Maintenance
PLANET4	Practical Learning of Artificial Intelligence on the Edge for indusTry 4.0
PLC	Programmable Logic Controller
PRZ	Rzeszów University of Technology
RUL	Remaining Useful Life
SCADA	Supervisory Control And Data Acquisition
SMEs	Small and Medium-sized Enterprises
UNIPI	University of Pisa
UOI	University of Ioannina
URL	Universitat Ramon Llull
VR	Virtual Reality

## Appendix A

The needs identified in industrial interviews:

**1-PRZ-C1**

The main identified needs are as follows:

- Developing the competencies of low-level staff to operate and use ICT systems—training and courses are needed to gain the ability to use ICT systems.
- Understanding the purpose and idea of Industry 4.0 by mid-level-staff—training and courses are needed to familiarize and understand the opportunities that Industry 4.0 technologies offer for specific areas of business operation and workplace efficiency increase.
- Acquisition of practical skills in using AI tools and methods by senior-level staff—in addition to seminars, discussions, and expert panels that help raise knowledge, training and practical courses related to specific tools and software are needed.
- Soft competencies—raising awareness of the need for Industrial 4.0 implementation in the company and building a culture of active participation in the implementation of ICT and Industry 4.0 technologies in the company is indispensable.
- Development of employees’ competencies in data engineering and data science.
- Support for human personnel by AI systems in data analysis and decision-making.
- Improving ICT and Industry 4.0 systems implementation process—quality and efficiency using standards and unified platforms.
- Advanced predictive maintenance systems, and systems for diagnosis and supervision of technological processes.
- Automated intralogistics systems.
- Automated systems for machines retooling with intelligent decision support.

**2-PRZ-C2**

The main identified needs are as follows:

- Development of knowledge and practical skills among employees in supplying AI methods and Industry 4.0 technology—in addition to seminars, discussions, and expert panels that help raise knowledge, training and practical courses related to specific tools and software are needed.
- Development of employees’ soft competencies such as: involvement, thinking logically and working as a team.
- Development of employees’ competencies in data engineering and data science.
- Improving ICT and Industry 4.0 systems implementation process—quality and efficiency using standards and unified platforms.
- Use of AI systems in data analysis and support for human employees in decision-making in products quality area—quality assurance.
- Advanced predictive maintenance systems, and systems for diagnosis and supervision of technological processes.

**3-PRZ-C3**

The main identified needs are as follows:

- Development of knowledge and practical skills among employees in applying AI methods and Industry 4.0 technology—in addition to seminars, discussions, and expert panels that help raise knowledge, training and practical courses related to specific tools and software are needed.
- Development of employees’ competencies in data engineering and data science: automated dashboards, automated reports.
- Improving ICT and Industry 4.0 systems implementation process—quality and efficiency using standards and unified platforms for data collection and integration.
- Application of AI methods to prediction of faculty results at the end of the month and engine behavior during testing.
- Use of AI systems in data analysis and support for human employees in decision-making in current production management.

**4-PRZ-C4**

The main identified needs are as follows:

- Development of knowledge and practical skills among employees in applying AI methods and Industry 4.0 technology—in addition to seminars, discussions, and expert panels that help raise knowledge, training and practical courses related to specific tools and software are needed.
- Development of employees’ competencies in data engineering and data science: automated dashboards, automated reports.
- Development of employees’ competencies in Lean manufacturing, production understanding—OEE, etc., running projects using project management methods.
- Improving ICT and Industry 4.0 systems implementation process—quality and efficiency using standards and unified platforms for data collection and integration.
- Use of AI systems in data analysis and support for human employees in decision-making in technological processes.
- Development of employees’ soft competencies, such as involvement, ability to think logically, ability to work as a team, interpersonal communication, self-motivation and self-organization, ability to establish relationships, decision making and perseverance.

**5-PRZ-C5**

The main identified needs are as follows:

- Implementation of self-management for two manufacturing lines operating on a supplier-customer basis.
- Implementation of automatic quality control in different areas.
- Implementation of AI in scheduling optimization.
- Implementation of automatic data acquisition.
- Implementation of data analytics.

The lacking competencies are as follow:

- Ability to identify areas where I4.0 solutions can be implemented.
- Knowledge about solutions that can be implemented.
- Knowledge about possible benefits of implementing I4.0 technologies, particularly economic savings—all expenses have to be justified.

**6-PRZ-C6**

The main needs identified in the company are as follows:

- The need to identify important manufacturing process parameters to be monitored and then analyzed to support the decision-making process.
- The need for systems integration.
- The need to implement a system supporting the planning process.
- The need to monitor the condition of machines.
- Need for data-driven cause-and-effect analysis.

**7-UNIPI-C1**

The main identified needs are as follows:

- Knowledge of AI capabilities and possible usage.
- Implementation of edge data processing to overcome customers’ scepticism about data streams.
- Implementation of local data analysis, eliminating the need to transmit data to a central office.
- Need for workers with more managerial skills, even at the cost of less specific expertise.

**8-UNIPI-C2**

The main identified needs are as follows:

- Standardization of data extraction,
- Standardization of data analysis,
- Knowledge and possible applications of EC,
- The need for employees with a broader view of data, from data collection to its application.

**9-UNIPI-C3**

Some employees are afraid of the Industry 4.0 revolution, robotics etc. The companies need to explain the advantages of I4.0.

**10-UNIPI-C4**

The company needs more students to implement IoT systems and I4.0 solutions on customers’ machines. Students and employers are not ready to work on industrial IoT systems.

**11-UNIPI-C5**

The main identified needs are as follows:

- Ability to revamp older machines, allowing them to collect data.
- Ability to analyze the data captured from machines, monitoring different parameters necessary for compliance with current regulations.
- Knowledge of AI systems for taking decisions or helping human figures in detecting anomalies that need an intervention.
- Improvement of current professional figures, teaching them new technologies and their potential.

**12-URL-C1**

The main identified needs are as follows:

- Getting the correct quantity and quality of data (Guidelines for data collection).
- Guidelines for identifying the right technology for the right application scenarios for IoT.
- Data security, powering of EC entities, administration of these entities and in overall the retrofitting of all these systems are required for their success.
- Workers with the knowledge of the latest technologies together with embedded systems.

**13-URL-C2**

The main identified needs are as follows:

- Development of I4.0 for product selling.
- Implementation of Business Intelligence for the Customers Journey.
- Implementation of a more integrable antenna (plug & play) for IoT products.
- Employees with competencies in hardware and software as well as engineers with competencies in “market adoption” are needed.
- Employees with strong competencies in electronics, antennas and Radio Frequency, some knowledge of the wireless industry, radio protocols and their applications, English, and soft skills such as being a good listener, creative, etc., are needed.

**14-URL-C3**

The main identified needs are as follows:

- Implementation of I4.0 for producing polyester for swimming pools, which requires connecting machines, connecting the plants, having automated (interrelated) data and personalized robots (machines are not standardized).
- Implementation of IoT for machines maintenance (vibrations, etc.), emissions, and humidity.
- Implementation of automated processes using robots.
- Implementation of a centralized application/database for all the data (production data, production studies, quality data, etc.) of the MES; consumes, waste management, optimization, acetone distribution acetone, KPIs from PowerBI, etc.
- Digitalization of the complete data gathering process (still, some parts are performed with a pen and paper).
- Need for a standardized ERP.
- Effective implementation of MES. Current solutions lack adaptability to internal processes. They are focused on automation companies, mono-product, and mono-phase. In the case of different products and different phases, important changes have to be made (in terms of coding and for workers). The workers feel under surveillance, and they do not like it.
- Keep staff updated on the latest technologies by enabling them to participate in robot courses (by robot selling companies) and in exhibitions.
- Employees with programming and network skills and strong knowledge in automation for implementing Industry 4.0.

**15-URL-C4**

The main identified needs are as follow:

- Need for data science and data management skills.
- ERP system had to be adapted. They offer the tailoring of the ERP as a product for other companies.
- The mass-warning systems could incorporate sensors. Currently, the company has skills in that neither.

**16-URL-C5**

The main identified needs are as follows:

- Implementation of I4.0 for car production by connecting all production components from all production sites in the cloud, using cross-data analysis to optimize production and logistics, and creating information for management for decision making.
- Implementation of AI to improve production machines.
- Implementation of Machine Learning for robots and cars maintenance.
- Implementation of aggregated statistics (up to now, they are segregated by markets with different CRMs).
- There is a need for employees with knowledge in data analytics, AI and machine learning combined with business. Technologic solutions should be proposed based on the needs/strategy of the company. Studies should unify business world with software world and industry (agile), or fill the gap between them. Engineers should have more knowledge on business.

**17-UOI-C1**

The basic needs identified during the interview can be summarized as follows:

- The need for securing the company’s network with modern technologies, as the company is vulnerable to cyber-attacks: The hard recovery after undergoing relevant experiences in the past has proven the need for adopting and integrating advanced security systems in the company’s networks.
- The exploitation of Virtual Reality technology in the direction of conducting simulations useful for the company’s engineers to test their designs and drawings.
- The synchronization of production lines by using robotic arms that bear intelligence and be capable of functioning autonomously.

**18-UOI-C2**

The main identified needs are as follows:

- Demand for automation in sharing and transferring data with a customer.
- Need for replacing old-fashioned systems that cannot adapt to the new era because compatibility issues emerge between different APIs.
- Developing state-of-the-art ERP systems that can cater to the company’s needs and tackle compatibility issues.
- Need for more high-skilled analytics experts.
- Need for people that could function as the liaison between the researcher on the domain of data analytics and the final product. A person that would understand both sides: the state-of-the-art in data analytics and the customer’s demands.
- Familiarization of all the employees with the cloud platforms. Employees should be flexible to work on any platform seamlessly.

**19-UOI-C3**

The main identified needs are as follows:

- Development of ERP systems that are not just logistics tools but embrace AI technologies.
- The employees should know to program and understand how this process works. They should have a general perception of the process, know to code and be able to comply with a specific methodology.

**20-UOI-C4**

The main identified needs are as follows:

- Need to invest (money and time) in ERP systems that provide a wide range of capabilities (not just financial management).
- Be trained on apprehending helpful information. Be familiar with extracting meaningful information from a vast amount of data.
- Employees should be adequately educated on the basics, have a good perception of the problems/tasks they undertake, be able to find solutions on their own, and should have the quality of lifelong self-learning. Moreover, the ability for problem-solving and critical thinking are indeed major assets for an employee.
